# The peripheral blood proteome signature of idiopathic pulmonary fibrosis is distinct from normal and is associated with novel immunological processes

**DOI:** 10.1038/srep46560

**Published:** 2017-04-25

**Authors:** David N. O’Dwyer, Katy C. Norman, Meng Xia, Yong Huang, Stephen J. Gurczynski, Shanna L. Ashley, Eric S. White, Kevin R. Flaherty, Fernando J. Martinez, Susan Murray, Imre Noth, Kelly B. Arnold, Bethany B. Moore

**Affiliations:** 1 Division of Pulmonary and Critical Care Medicine, Department of Internal Medicine, University of Michigan, Ann Arbor, MI, USA; 2 Department of Biomedical Engineering, University of Michigan, Ann Arbor, MI, USA; 3 Biostatistics Department, University of Michigan School of Public Health, Ann Arbor, MI, USA; 4 Section of Pulmonary and Critical Care Medicine, University of Chicago, Chicago, IL, USA; 5 Immunology Graduate Program, University of Michigan, Ann Arbor, MI, USA; 6 Department of Internal Medicine, Weill Cornell Medical College, New York, NY, USA; 7 Department of Microbiology and Immunology, University of Michigan, Ann Arbor, MI, USA

## Abstract

Idiopathic pulmonary fibrosis (IPF) is a progressive and fatal interstitial pneumonia. The disease pathophysiology is poorly understood and the etiology remains unclear. Recent advances have generated new therapies and improved knowledge of the natural history of IPF. These gains have been brokered by advances in technology and improved insight into the role of various genes in mediating disease, but gene expression and protein levels do not always correlate. Thus, in this paper we apply a novel large scale high throughput aptamer approach to identify more than 1100 proteins in the peripheral blood of well-characterized IPF patients and normal volunteers. We use systems biology approaches to identify a unique IPF proteome signature and give insight into biological processes driving IPF. We found IPF plasma to be altered and enriched for proteins involved in defense response, wound healing and protein phosphorylation when compared to normal human plasma. Analysis also revealed a minimal protein signature that differentiated IPF patients from normal controls, which may allow for accurate diagnosis of IPF based on easily-accessible peripheral blood. This report introduces large scale unbiased protein discovery analysis to IPF and describes distinct biological processes that further inform disease biology.

Idiopathic Pulmonary Fibrosis (IPF) is the most common idiopathic interstitial pneumonia and is a fatal progressive disease with a median survival of 2 to 3 years^1^. The etiology of IPF remains unclear and despite recent advances in therapy, IPF persists as an incurable disease^2^,^3^. IPF is characterized by certain clinical features with radiological and histopathological findings of usual interstitial pneumonia^1^. The disease results in progressive fibrotic remodeling of the pulmonary parenchyma with loss of structural integrity, impaired gas exchange and respiratory failure. The pathophysiology of IPF features a paradigm that involves injury, loss of the epithelial cell barrier with aberrant re-epithelialization, fibroblast activation and unregulated myofibroblast deposition of extracellular matrix components^4^.

The natural history of IPF is variable and patients can experience different and dynamic clinical courses with phenotypes ranging from accelerated disease with early mortality to slowly progressive disease^5^. Considerable resources have been employed to facilitate prediction and early identification of these phenotypes to improve transplantation strategies and the selection of appropriate patients for therapeutic trials. Studies have identified proteins and chemokines that may discriminate between disease phenotypes and predict clinical outcomes^6^,^7^,^8^. Several genomic expression profiles have reported associations with disease progression in IPF^9^,^10^ and the peripheral blood transcriptome may discriminate between mild and severe disease graded by diffusion capacity^11^. Genetic risk loci include single nucleotide polymorphisms in the Toll interacting (TOLLIP) gene, toll like receptor (TLR) 3 gene and MUC5B promoter^12^,^13^,^14^. These key advances have elucidated new potential mechanisms and therapeutic targets and have advanced the role of “omics” in IPF. However, a greater understanding of the relationship between genomic risks and the mechanistic impact on IPF pathophysiology is required. For instance, disease susceptibility is increased by the MUC5B polymorphism yet survival is improved^15^. The genome is subject to post transcriptional manipulation by micro-RNA (miRNA). Altered levels of miR-200 and miR-21 have reported associations with fibrogenesis in experimental models and human IPF patients^16^,^17^. Furthermore, circulating miRNA’s have been found in the blood of IPF patients and several miRNAs are differentially expressed in rapidly progressive disease^18^. Micro-RNA may act as regulators of disease progression and therefore the transcriptome and genome may be subject to significant modifications in IPF. An accurate “snapshot” of disease biology may require analysis of protein or the “proteome” in IPF patients. IPF is heterogeneous with distinct individual variation in the clinical courses that patients encounter. It is plausible that distinct and dynamic biological processes manifest as a common clinical phenotype, as evidenced by the UIP pattern on histopathology and imaging. The application of a new approach focused on identifying these processes or “molecular endotypes” may facilitate improved understanding of disease biology, molecular pathways and the mechanisms behind the IPF clinical phenotypes^19^,^20^.

Studies of the IPF proteome to date have focused on bronchoalveolar lavage fluid (BALF) and lung tissue analysis^21^,^22^,^23^,^24^. Novel targets have been reported including CCL24^21^, and putative molecular pathways have been identified including the unfolded protein response through proteomic studies^22^. While BALF may be desirable for analysis given it is an accessible component of the lung environment, it is acquired through an invasive endoscopic procedure and subject to variability in representative sampling and processing. Furthermore, many patients may be unable to undergo the sampling procedure; thus, accurate analyses from peripheral blood would be optimal for patients. New proteomic assays have been developed that utilize modified aptamers termed SOMAmers^©^ (slow off rate modified aptamers)^25^. This assay can readily analyze over 1,000 proteins at varying levels of abundance in the peripheral blood. The SOMAmer^©^ platform has been employed in biomarker discovery in several diseases to date^26^,^27^,^28^,^29^,^30^. We have previously published a panel of 6 SOMAmer^©^ measured proteins which accurately predicts disease progression in IPF^31^. In this paper, we apply for the first time, aptamer technology to identify on a large scale the differentially expressed proteins in the blood of IPF patients compared to normal controls. We then use this information to describe in detail the biological processes and molecular pathways that may discriminate the disease biology of IPF. The ultimate goal of this work is not to identify or validate particular proteins as biomarkers, but rather to understand what biological pathways are aberrant in IPF vs. control patients based on the peripheral blood proteome.

## Results

### The peripheral proteome of IPF patients is distinct from controls

**Figure 1:**
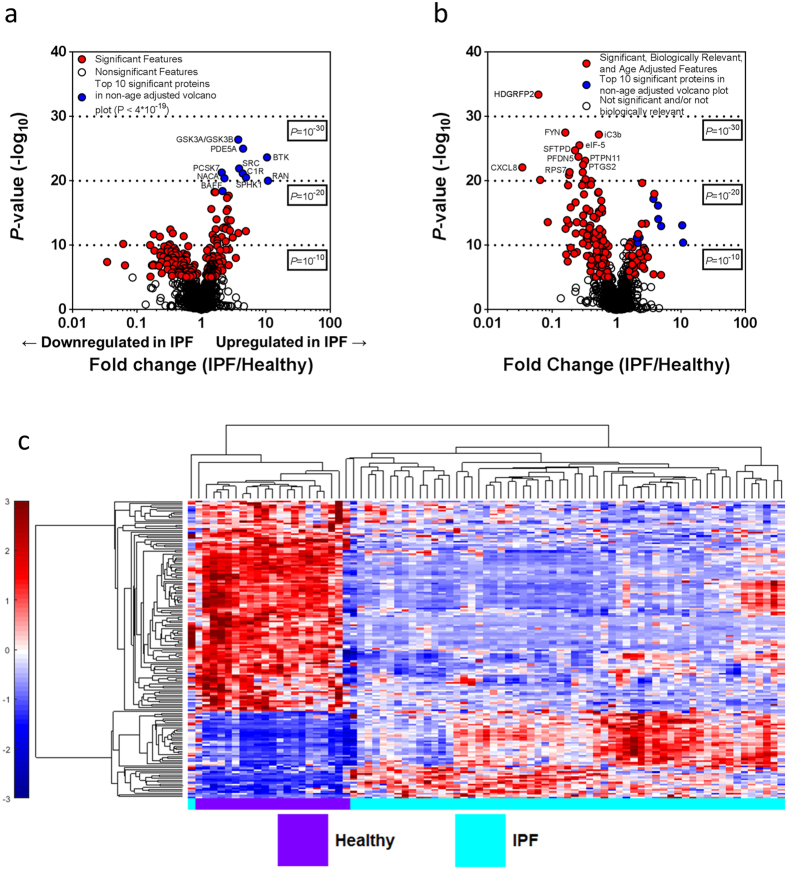
The peripheral plasma in IPF is distinct from normal controls. (**a**) Volcano plots highlighting fold change (x axis) and the significance level on the y axis of the blood proteins measured in the SOMAmer Aptamer assay in the COMET study. Points in red indicate proteins that are significantly different in the healthy versus IPF patients when correcting for multiple comparisons using the Bonferroni method with a corrected P-value of 0.01. Points in blue are the top ten most significant proteins when age is not considered. (**b**) Volcano plot with age adjustment. Points in red indicate proteins that are significantly different between healthy and IPF patients when adjusted for the age difference between the two groups and when correcting for multiple comparisons using the Bonferroni method with a corrected P-value of 0.01. The points in blue are the same as in panel “a”. (**c**) Hierarchical clustering of age-adjusted blood proteins that were determined to be significantly different and biologically relevant between healthy and IPF patients shows visually distinct blood proteomes between healthy and IPF patients. With the exception of two individuals, this protein signature in the blood was able to perfectly differentiate between healthy and IPF patients. The abundance of each protein is shown in color, with red meaning overabundant proteins, white unchanged, and blue being underabundant proteins, all compared to the mean (color bar scale is to the left of figure). Hierarchical clustering of proteins was generated by unsupervised average linkage using Pearson’s correlation as the distance metric.

The demographics and clinical characteristics of study subjects are summarized in Supplementary Table S1. This population of IPF patients was a sub cohort of the COMET trial. The initial proteomic analysis included all 1129 available analytes which span a wide variety of biological processes and molecular pathways. Relevant comorbidities are reported in Supplementary Table S2. We applied analysis (see schematic in Supplementary Figure S1) to the blood proteins measured in the SOMAscan assay in order to find differences in the blood protein profiles of healthy and fibrotic patients. From a total of 1129 plasma proteins, 203 were found to have a mean value that was significantly different (both upregulated and downregulated) than the mean value of the same analyte in control patients, with a Bonferroni corrected α of 1% (*P* < 0.0000089) (Fig. 1a). The top 10 significantly different values (all significant after Bonferroni correction with *P* < 4E-19) included glycogen synthase kinase-3 alpha/beta (GSK3A/GSK3B; 3.73 fold change), proto-oncogene tyrosine-protein kinase Src (SRC; 3.85 fold change), complement C1r subcomponent (C1R; 4.39 fold change), Proprotein convertase subtilisin/kexin type 7 (PCSK7; fold change 2.07), cGMP-specific 3′,5′-cyclic phosphodiesterase (PDE5A; 4.44 fold change), sphingosine kinase 1 (SPHK1; 4.92 fold change), tyrosine-protein kinase BTK (BTK; 10.45 fold change), B-cell activating factor (BAFF; fold change 2.13), nascent polypeptide-associated complex subunit alpha (NACA; 2.28 fold change), and GTP-binding nuclear protein Ran (RAN; 10.78 fold change). Interestingly, these 10 proteins that were most significantly different between control and IPF patients were all increased in the IPF patients.

We next applied a secondary method to account for age differences between control and IPF cohorts. This screen identified 48 proteins which were expressed at significantly elevated or upregulated levels (≥1.5 fold) in the blood of IPF patients at screening when compared to controls (Supplementary Table S3). This represents 4.3% of total screened analytes. The screening process further identified 116 proteins which were expressed at significantly reduced or downregulated levels (≤0.75 fold) in the blood of IPF patients when compared to controls (Supplementary Table S4). This represents 10.3% of the screened analytes. A list of all significant proteins with their fold expression is reported in Supplementary Table S5. These biologically relevant, age-adjusted, significantly different proteins were then highlighted in a volcano plot (Fig. 1b). The top ten significantly different, age-adjusted proteins were hepatoma-derived growth factor-related protein 2 (HDGFRP2; fold change 0.06), inactivated complement 3b (iC3b; fold change 0.53), tyrosine-protein kinase FYN (FYN; fold change 0.16), pulmonary surfactant-associated protein D (SFTPD; fold change 0.23), eukaryotic translation initiation factor 5 (EIF5; fold change 0.26), prefoldin subunit 5 (PFDN5; fold change 0.25), tyrosine-protein phosphatase non-receptor type 11 (PTPN11; fold change 0.33), prostaglandin G/H synthase 2 (PTGS2; fold change 0.30) 40S ribosomal protein S7 (RPS7; fold change 0.19), interleukin-8 (IL8; fold change 0.034). Interestingly, when the effects of age were addressed when performing the t-tests, the top ten significantly different proteins were all increased in healthy patients.

To better visualize how this age-adjusted, biologically relevant protein signature differentiated the two groups, we performed hierarchical clustering on the 48 upregulated and the 116 downregulated, age-adjusted, significantly different proteins (identified in Fig. 1b) between healthy and IPF patients. The result was almost ideal differentiation of the healthy and IPF groups (Fig. 1c). Overall this analysis indicated visually distinct proteomes could be measured in healthy and IPF patients using a subset of 164 analytes within the SOMAscan Assay®.

The two most common co-morbidities in this patient cohort were gastroseophageal reflux disease (GERD) and obstructive sleep apnea (OSA) (Supplementary Table S2). Principal component analysis demonstrates that the greatest differences in the proteomic data arise from variation between the healthy and IPF groups, with no apparent clustering due to the co-morbidities (Supplementary Figure S2). Comorbidity information was not available for the healthy controls.

### Enrichment and network analysis of the upregulated IPF plasma proteome

**Figure 2:**
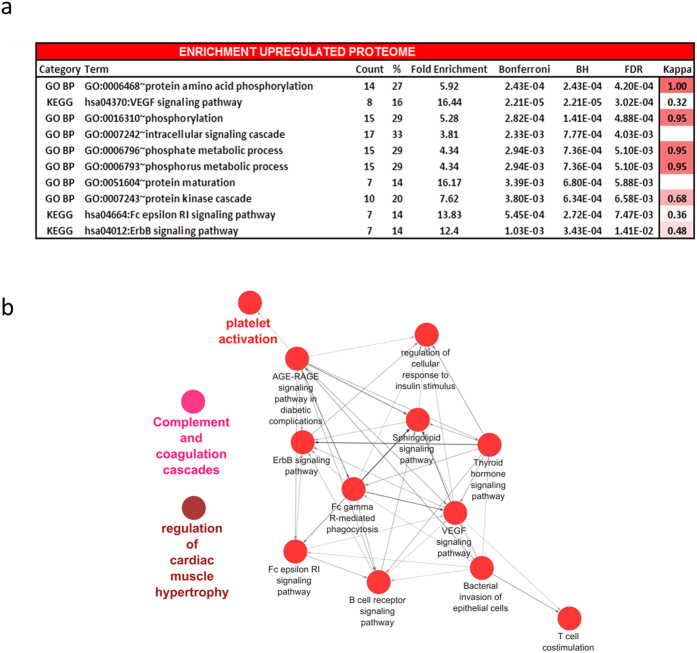
Enrichment and network analysis of the upregulated IPF plasma proteome. (**a**) DAVID enrichment analysis was employed to select the most significantly enriched terms within the sample of upregulated proteins (n = 48). Bonferroni corrected P value, Benjamini-Hochberg (BH) P value and False Discovery Rates (FDR) are reported. Kappa statistics reporting similarity to most significant term (low > 0.25, moderate 0.25–0.5, high 0.5–0.75, very high 0.75–1). (**b**) ClueGO visualization and analysis of biological role (GO, Kegg pathways) was undertaken. GO terms are mapped in clusters by Kappa statistics. [Hexagon = Kegg pathway, Ellipse = Gene ontology term, arrow depicts direction of association].The major overview term (smallest P value within cluster) is depicted in color. Node size depicts Bonferroni corrected P value < 0.0005 for all terms reported. Further details can be found in online supplement.

The next step was to utilize our differentially expressed proteins to gain systems level insight into the disease biology of IPF. This was achieved through enrichment analysis using the online DAVID software tool. DAVID associates proteins to hierarchically clustered functional terms (Gene Ontology, Kegg Pathway), and an enrichment score is calculated. The most significantly enriched processes included protein amino acid phosphorylation, VEGF signaling and intracellular signaling cascade (see Fig. 2a).

We next looked at possible networks and relationships between these proteins using the ClueGo application in Cytoscape. Proteins are clustered within enriched terms (Gene Ontology, Kegg Pathway) and the degree of similarity between clusters is calculated using Kappa statistics. The significantly enriched clusters included platelet activation (P = 17.0E-12), the regulation of cardiac muscle hypertrophy (P = 2.9E-6) and complement and coagulation cascades (P = 53.0E-6) (Fig. 2b). The level of agreement between each cluster and term is reported by Kappa statistics (Supplementary Fig S3). Statistical values for each reported term are listed in Supplementary Table S6. In order to biologically validate our proteomic pathway discovery findings, we analyzed threshold values of transcriptomic data from peripheral blood cells in the same patients and report that VEGF-related genes correlate with VEGF -related proteins as measured by aptamers (data not shown). These differentially expressed VEGF -related genes when analyzed by Kegg pathway are enriched in biological pathways that are plausibly related to VEGF signaling, providing biological validation of our findings.

### Enrichment and network analysis of the downregulated IPF plasma proteome

**Figure 3:**
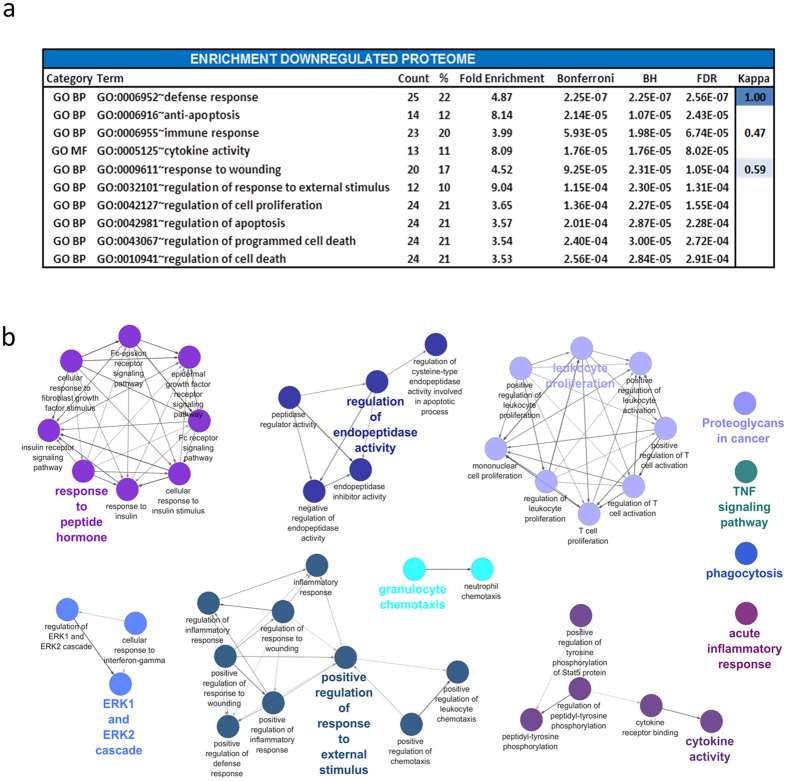
Enrichment and network analysis of the downregulated IPF plasma proteome. (**a**) DAVID enrichment analysis was employed to select the most significantly enriched terms within the sample of downregulated proteins (n = 116). Bonferroni corrected P value, BH P value and FDRs are reported. Kappa statistics reporting similarity to most significant term (low > 0.25, moderate 0.25–0.5, high 0.5–0.75, very high 0.75–1). (**b**) ClueGO visualization and analysis of biological role (GO, Kegg pathways) was undertaken. GO terms are mapped in clusters by Kappa statistics. [Hexagon = Kegg pathway, Ellipse = Gene ontology term, arrow depicts direction of association].The major overview term (smallest P value within cluster) is depicted in color. Node size depicts Bonferroni corrected P value < 0.0005 for all terms reported. Further details can be found in online supplement.

The downregulated proteins were analyzed for enrichment using the DAVID online software tool. The most significantly enriched terms (GO ontology, Kegg pathway) included defense response, anti-apoptosis and immune response (see Fig. 3a). Cytoscape and ClueGo were then utilized to examine possible networks and relationships between enriched terms and their associated proteins. These significant clusters included acute inflammatory response (P = 740.0E-9), response to peptide hormone (P = 3.4E-15), phagocytosis (P = 1.8E-6), regulation of endopeptidase activity (P = 14.0E-12), leukocyte proliferation (P = 25.0E-9), ERK1/2 cascades (P = 150.0E-12), granulocyte chemotaxis (P = 22.0E-9), positive regulation of a response to an external stimulus (P = 74.0E-24), TNF signaling pathway (P = 4.2E-6), proteoglycans in cancer (P = 530.0E-9) and cytokine activity (P = 140.0E-15) (Fig. 3b). Kappa statistics for similarity between gene, terms and clusters can be found in Supplementary Figure S4. Statistical values for each reported term are listed in Supplementary Table S7.

### A unique protein signature involved in immune processes differentiates IPF patients from controls

**Figure 4:**
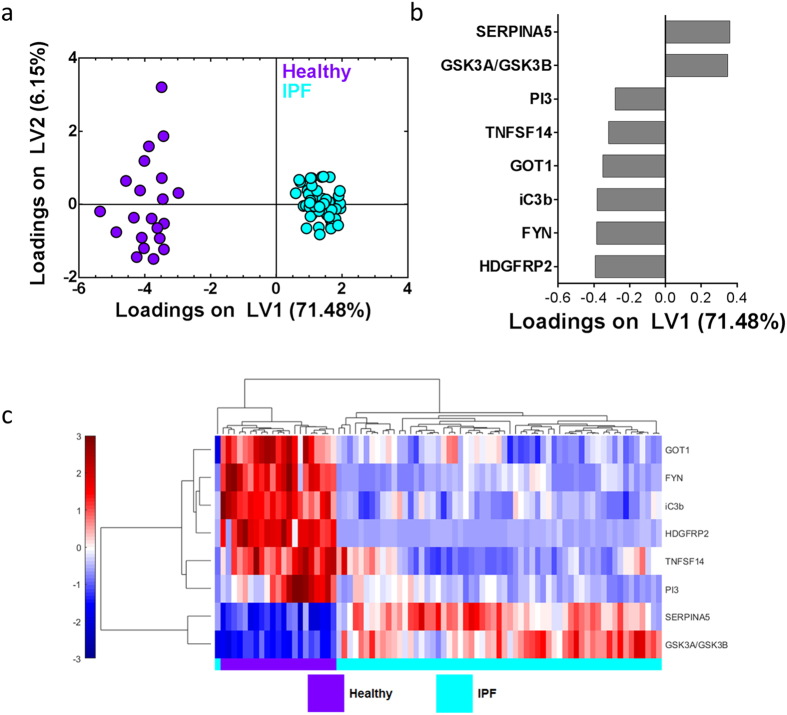
LASSO/PLSDA identified a minimum protein signature of 8 age-adjusted proteins that best differentiated healthy and IPF patients. (**a**) LASSO identified an 8-protein signature that differentiated healthy (purple) and IPF (cyan) patients, with 100% calibration accuracy and 100% cross-validation accuracy, with 100% sensitivity and specificity for both healthy and IPF patients. Latent variable 1 (LV1) accounted for 71.48% of the variance in the data, and latent variable 2 (LV2) accounted for 6.15% of the variance in the data. (**b**) The loadings plot indicates protein contributions to the LASSO-identified signature, with positive loadings positively associated with IPF, and negative loadings comparatively reduced in IPF. (**c**) Hierarchical clustering further emphasizes the visual difference between healthy and IPF patients based on the LASSO-identified signature. Abundance of each protein is shown in color, with red indicating overabundance, white unchanged, and blue indicating underabundant proteins compared to the mean. Color bar scale is to the left of figure.

We next wanted to find a minimum set of proteins that best differentiated the healthy and IPF patients based on covariance, or relationships between proteins. This signature could potentially be used as a diagnostic tool based on non-invasive measurements made from peripheral blood. To identify the minimum multivariate protein signature that differentiated healthy and IPF patients, we used the Least Absolute Shrinkage and Selection Operator (LASSO) method as a feature selection tool, followed by Partial Least Squares Determinant Analysis (PLSDA) to assess the usefulness of the identified signature. LASSO identified an age-adjusted signature of 8 proteins that best differentiated the healthy patients from the patients with IPF. A PLSDA model of these 8 selected proteins classified the two groups perfectly, with 100% calibration accuracy and 100% cross-validation accuracy, as well as 100% sensitivity and specificity for both the healthy and the IPF groups. Latent variable 1 (LV1) was able to completely differentiate between healthy patients (negative scores on LV1) and patients with IPF (positive scores on LV1; Fig. 4a). Two of the 8 proteins were loaded positively on LV1 (Fig. 4b), indicating that they were positively associated with the IPF patients, whereas 6 proteins were loaded negatively on LV1, indicating that they were negatively associated with the IPF patients (Fig. 4b). Not surprisingly, all of the proteins identified by LASSO were also found to be significantly different between healthy and IPF patients in the volcano plot (Fig. 1b). LASSO and PLSDA were able to successfully separate individuals that were healthy from individuals with IPF; this suggests that the 8 proteins in the signature may have relationships that are of biological interest. The LASSO-signature does include proteins that have clear immunological functions: inactivated (iC3b) and tumor necrosis factor ligand superfamily member 14 (TNFSF14 or LIGHT). This further suggests the potential importance of immune processes in the pathogenesis of IPF and warrants further investigation.

In order to better visualize patient clustering using our LASSO-identified signature, we performed hierarchical clustering and created a heat map of the LASSO-identified protein signature (Fig. 4c). The result was readily-identifiable, near-perfect clustering of the healthy and IPF patients, with only one patient being misclassified. Interestingly, the 2 proteins in the hierarchical cluster that were overabundant in the IPF patients are the same 2 proteins that PLSDA identified as being positively associated with the IPF patients. Recalling that all 8 of the proteins were also included in the biologically relevant, age-adjusted significantly different protein panel, these findings validate the LASSO-identified blood protein signature as being the preferred signature to differentiate the two groups of patients, and also support the idea that there are large differences in the blood proteome seen in healthy and IPF patients. We also analyzed the LASSO-identified protein signature using GO terms for biological process and molecular function. The most significantly upregulated functional annotation cluster involved peptidase inhibitors, endopeptidase regulators and catalytic activity (FE = 3.46, Bonferroni corrected P value = 0.0135) (Supplementary Figure S5). Overall these results provide proof-of-concept and suggest value for these approaches in the future development of a non-invasive diagnostic assay for IPF. This could be especially useful for a diagnosis of IPF with relatively normal pulmonary function levels and/or atypical radiological findings.

## Discussion

IPF remains a disease of unknown etiology with poorly understood pathophysiological mechanisms. Major advances have occurred in recent years through hypothesis-driven studies of potential biomarkers of the genome, transcriptome, chemokines and cytokines. In this paper we apply novel modified aptamer technology to produce large scale studies of proteins of variable abundance in the blood of IPF patients and normal controls for the first time. This novel approach to IPF has generated new hypothesis-provoking insight regarding the possible key functional biological pathway abnormalities in IPF. The design and main focus of this study was to identify differentially expressed proteins in the blood of IPF patients compared to normal healthy controls and through the employment of systems biology and bioinformatics tools, generate knowledge about the enriched biological processes that these proteins may represent.

Analysis of the downregulated protein profile identifies a role for defense response encompassing a reaction to the presence of a foreign body or injury with an associated attempt to restrict damage and initiate repair. This is the most significantly enriched process within the downregulated protein panel. These data suggest that compared to a normal host, IPF patients have reduced levels of circulating proteins that support host defense. Indeed, the cohort of patients studied in this work (COMET study cohort) was previously employed in a project that supported a role for dysbiosis in the lung and disease progression. Alterations in the microbiome, namely an increase in *Streptococcal* and *Staphylococcal* operational taxonomic units were associated with disease progression in IPF^32^. Molyneaux *et al*. have reported an association between disease progression and increased bacterial burden in the lung^33^. An increased quantity of *Streptococcus* species was noted. Knippenberg *et al*. using murine models have demonstrated a mechanism by which a pneumococcal toxin, pneumolysin, exacerbates pulmonary fibrosis^34^. Our study of the proteome at trial screening suggesting a reduction in processes supporting host defense, supports a potential role for pathogens, particularly given further findings in the downregulated proteome involving the regulation of responses to external stimuli. These data enrich the evidence for a potential role for dysbiosis in IPF progression.

Features of acute inflammation including leucocyte chemotaxis, proliferation and phagocytosis are subject to downregulation in the blood compared to normal controls in our study. Several proteins involved in regulating the response to wounding appear inhibited in the plasma of patients with IPF compared to controls. We hypothesize that this finding is indicative of the recurrent injury and loss of the alveolar epithelial barrier. The proteome findings in this study support the paradigm of recurrent injury or wounding with aberrant repair. Indeed, our findings support an intrinsic impairment of the immune response to stimuli which may, in turn, promote insufficient or even exuberant responses to improve pathogen clearance but worsen bystander damage. The response of Toll like receptors (TLRs) and other pathogen recognition receptors to pathogen associated molecular patterns (PAMPs) and danger associated molecular patterns (DAMPs) is crucial to mounting a response to infection and injury^35^. IPF patients may have impaired responses to DAMPs and PAMPs. Studies of pathogen recognition receptors involved in responses to PAMPs/DAMPs including TLR 3 and TOLLIP have reported associations with IPF pathophysiology^13^,^14^. Furthermore, the role of immunosuppression is associated with poorer survival and higher levels of hospitalization in IPF patients^36^. The addition of agents responsible for attenuated immune responses may contribute negatively to a disease biology that features impaired responses to PAMPs and DAMPs.

The upregulated protein profile identified T cell co-stimulation as a process discriminating between normal and IPF patients. The role of T cell co-stimulation in regulation of lung fibrosis is controversial and complicated by the fact that measurements have been based on samples taken from different human compartments versus murine models. Studies to date have supported a role for decreased expression of inducible T cell co-stimulator (ICOS) in peripheral blood mononuclear cells (PBMCs) as a marker of disease progression and a predictor of poor survival outcomes^9^,^10^. However, animal models of bleomycin-induced pulmonary fibrosis reported higher levels of ICOS ligand (ICOSL) expression on macrophages and B cells in ICOS deficient mice compared to wild type which correlated with higher levels of fibrosis, thus highlighting a role for ICOSL expression in positively regulating pulmonary fibrosis. ICOS deficient mice had attenuated pulmonary fibrosis upon bleomycin challenge^37^. The role of ICOS and T cell co-stimulation warrants further study given our findings of enrichment of this process in the upregulated proteins when comparing IPF patients to normal controls. We have shown that ICOS may be secreted by activated T lymphocytes^31^ and hypothesize that the loss of ICOS expression on cells may correlate with elevated plasma levels and that this may be accompanied by reduced transcription. Taken together, these changes suggest a crucial regulatory step in the pathobiology of IPF. Interestingly, the positive regulation of T cell activation is notably enriched within the downregulated plasma proteome in IPF patients suggesting that overall, IPF patients may have impaired T cell activity and this may be linked to disease biology, potentially via impaired defense against pathogens such as herpesviruses^38^.

Protein phosphorylation is a fundamental mechanism of signal transduction and is achieved by kinase activity. The high signal for phosphorylation in our upregulated proteome may represent heightened kinase activity and both these processes are enriched within the upregulated proteome. *In vitro* studies and animal models have produced robust evidence to support a central role for protein kinase activity in pulmonary fibrosis, particularly tyrosine kinase activity including platelet derived growth factor (PDGF), epidermal growth factor (EGF), fibroblast growth factor (FGF) and vascular endothelial growth factor (VEGF)^39^. Nintedanib, a novel and approved tyrosine kinase inhibitor for IPF, robustly inhibits VEGF receptor, PDGF receptor and FGF receptor with resultant modification of IPF fibroblast biology and improved patients outcomes^3^,^40^,^41^. VEGF signaling was additionally enriched within the upregulated plasma proteome of IPF patients in our work, consolidating its role in IPF pathogenesis. A key downstream event of ligation between these tyrosine kinases and their receptors is autophosphorylation and phosphatidylinositide 3-kinase activity^42^,^43^. ErbB signaling enrichment is also notable. These are a family of tyrosine kinase receptors, which include Her1 (epidermal growth factor receptor (EGFR)), Her2, Her3 and Her4. Several of these receptors have reported roles in epithelial remodeling, epithelial proliferation and are found to play significant roles in models of fibrosis^44^,^45^,^46^. Further dysfunction within this pathway is supported by the finding of enrichment within the downregulated proteome for EGFR (Her1) signaling. EGFR is vital for normal epithelial repair so downregulation of this pathway could indicate impaired wound healing. Alternatively, we cannot rule out the possibility that EGFR signaling within the lung promotes fibrosis, but that the signature is lost in peripheral blood. Further investigation of the role of ErbB signaling in the pathogenesis of IPF is likely needed.

Platelet activation leads to the release of several profibrotic mediators and IPF patients have reported evidence of increased platelet reactivity and activation in a previous study^47^. It is possible that this is reflective of the IPF plasma environment. Complement and coagulation cascades have reported associations with IPF. Complement receptor polymorphisms may be associated with the development of IPF^48^. Furthermore, complement can augment epithelial injury in pulmonary fibrosis through crosstalk with Transforming Growth Factor-β (TGF-β)^49^. Gu *et al*. demonstrated that the inhibition of both complement component C3a and C5a receptors can lead to the arrest of fibrosis and may have therapeutic potential in IPF^50^. The enrichment within the plasma proteome of platelet activation and complement cascades is suggestive of ongoing injury that is detectable in the blood and will require further study.

The LASSO/PSLDA proteome signature we have identified includes novel proteins that have no previous reported associations with IPF. Armed with these target proteins however, it is interesting to speculate on their putative roles in pulmonary fibrosis. TNFSF14 (Tumor necrosis factor ligand superfamily member 14 or LIGHT) is an inflammatory molecule and a member of the TNF superfamily that our analysis also shows to be downregulated in IPF plasma compared to normal. Seemingly contradictory, the genetic deletion of LIGHT attenuates bleomycin-induced pulmonary fibrosis in animal models through the abolition of Thymic stromal lymphopoietin (TSLP) expression^51^. In addition, Herro *et al*. demonstrated that the administration of recombinant LIGHT to murine models produced features of fibrotic lung disease similar to the bleomycin fibrotic phenotype, via a TSLP-dependent mechanism. Human bronchial epithelial cells challenged with LIGHT *in vitro* generate TSLP production^51^. LIGHT appears to have potential as a regulator of fibrosis and its role in IPF requires further exploration. LIGHT can function as a mediator of herpes viral cell entry, hence its acronym Herpes Virus Entry Mediator (HVEM), and one may speculate a further mechanistic role for LIGHT in this context given the evolving roles of herpes virus in fibrotic lung disease exacerbations^38^, but it may be informative to compare circulating vs. tissue measurements. Glycogen synthase kinase-3 alpha/Glycogen synthase kinase-3 beta(beta (GSK3A/GSK3B) are negative regulators of glucose homeostasis, Wnt signaling and transcription factors, and this protein is positively associated with IPF. GSK3A/GSK3B inhibition in bleomycin-exposed mice has been shown to reduce alveolitis, lung fibrosis, and alveolar cell apoptosis^52^. GSK3A/GSK3B inhibition also decreased the production of monocyte chemoattractant protein-1 (MCP-1/CCL2) and tumor necrosis factor-α (TNF-α) by lung macrophages after bleomycin exposure in this study. Plasma serine protease inhibitor (SERPINA5), a molecule we find at elevated levels in IPF relative to control patients, has been shown to be upregulated in the intra-alveolar space of patients with interstitial lung diseases (IPF included), and is involved in the inhibition of fibrinolysis, especially in IPF^53^. A reduction in fibrinolysis causes more collagen, fibrin, and other extracellular matrix fibers to accumulate in the intra-alveolar space of these patients, leading to a stiffer lung and to formation of a matrix where fibroblasts can proliferate and release more collagen^54^.

The acquisition of a distinct signature in the blood proteome of IPF patients that allows for discrimination between IPF and healthy controls is a significant proof of concept discovery. While we recognize that a blood test is not necessary to diagnose IPF patients from healthy volunteers, our work suggests that this methodology could be employed to help diagnose IPF from other forms of chronic lung disease. This will require further validation with larger numbers of patients, and exploration in other chronic lung diseases to determine whether differential signatures are producible in similar diseases. If true, the potential for change in clinical practice is considerable. The use of peripheral blood to identify disease-specific signatures may result in obviating the need for biopsy in patients who present with imaging features that are not consistent with IPF or possibly improve diagnostic confidence in patients who are not suitable for a surgical biopsy. Previous studies of plasma proteins in IPF patients identified both MMP-7 and MMP-1 as predictors of disease progression that were differentially expressed compared to normal plasma^8^. While there remain significant methodological differences between studies, we have found that MMP-7 is also upregulated in IPF plasma compared to normal.

There are several limitations to our study. The study numbers are limited and the IPF cohort, while extensively characterized, was not subject to death over the course of 80 week follow up. This population may not be fully representative of the IPF disease spectrum and we are not able to adjust for all potential confounding variables including co-morbidities within the IPF population. The absence of a validation cohort is a weakness; however the main goal of this work was to generate hypotheses based on the proteomic data accrued. The use of slow off rate modified aptamers is novel and the aptamer results may not correlate with other protein measurement platforms. The aptamers bind to non-linear sequences with very high specificity for the selected target; this may explain some of the variance when measuring identical targets with other platforms such as ELISA^25^. However, several studies have demonstrated very high levels of agreement between the modified aptamer platform and ELISA^31^,^55^.

Although we did not have a validation cohort to test the accuracy of our PLSDA model, we did investigate model accuracy through cross-validation. This involved excluding a small portion of the data (called the test set), building a model based on the rest of the data, and testing the accuracy of the model using the test set. By repeating this process many times and using different test sets, we were able to obtain the cross-validation accuracy by averaging the accuracy of each individual model. Thus despite the fact that there was not a validation cohort, we were still able to report a metric of model accuracy, which was calculated based on testing the model with unseen data. The final model we have reported on performed perfectly during cross-validation testing with 100% cross-validation accuracy.

Our work identified biological processes that discriminate IPF from healthy controls and generates hypothesis and new targets for investigation into disease mechanisms. Our study patients were recruited to a clinical trial with the highest standards of diagnostic approach and management. The prime purpose of this work was to introduce the approach of large scale unbiased biomarker screening and the generation of subsequent mechanistic hypothesis. However, given the proposed single organ nature of IPF, the biological signal detectable in blood is dilute and may not accurately reflect ongoing change within the lung. However, peripheral blood has been employed in several biomarker studies in IPF to date^6^,^8^,^10^ and represents an easily-accessible compartment for analysis. The fact that the identified proteome clustered differently between IPF and controls gives some confidence that analyses of peripheral blood may be useful.

In conclusion, this work furthers the evolving evidence supporting impaired host defense as a key marker of IPF disease biology and validates some of our current understanding. We generate further hypotheses about novel potential therapeutic targets and introduce a new approach to biomarker studies in IPF. The ability to identify a minimal signature that allows clinicians and researchers alike to discriminate IPF cases from normal serves as a proof of principle that this approach may have potential in defining other forms of chronic interstitial lung disease and the further evaluation of molecular endotyping in pulmonary fibrosis.

## Methods

### Study Population

Subjects included in this analysis were a subset of patients who participated in a prospective observational study correlating biomarkers with disease progression (clinicaltrials.gov, clinical trials ID no. NCT01071707) (Correlating Outcomes with biochemical Markers to Estimate Time-progression in Idiopathic Pulmonary Fibrosis–COMET). This cohort consisted of 60 patients who had samples available for analysis for at least 3 follow up time points, but this report focuses only on the baseline samples. Inclusion criteria required patients to be aged 35-80 years with a diagnosis of IPF. Exclusion criteria included a diagnosis of IPF that was > 4 years prior to screening, a diagnosis of collagen-vascular disorder, FEV1/FVC < 0.6, evidence of active infection at screening, or comorbid conditions other than IPF likely to result in death within one year. Subject follow up was for 80 weeks. Informed consent was obtained from all participating patients. The study protocol was reviewed and approved by the institutional review board of each participating center and methods were carried out in accordance with the relevant guidelines and regulations. Participating centers included: University of California Los Angeles. Los Angeles, CA, United States–University of California, San Francisco. San Francisco, CA, United States–National Jewish Medical and Research Center, Denver, CO, United States–University of Chicago, Chicago, IL, United States–University of Michigan Ann Arbor, MI, United States–Cleveland Clinic Foundation, Cleveland, OH, United States–Temple University, Philadelphia, PA, United States–Brown University, Providence, RI, United States–Vanderbilt University, Nashville, TN, United States. Patients were enrolled from March 2010 to March 2011. Blood samples and demographic data were also acquired from healthy human controls (n = 21). Demographics are displayed separately for IPF patients and healthy normal participants, with mean and standard deviation for the continuous predictor age and the number and percentage enrolled for the categorical variable gender. Statistical significance of differences between the two groups of people for age and gender were assessed via Student’s t test and Pearson’s Chi-squared test, respectively (Supplementary Table S1). Patients were diagnosed as having IPF using a multidisciplinary approach as per published international guidelines^1^. In brief, the diagnosis of IPF was on the basis of features on computed tomography (CT) scans of the chest or usual interstitial pneumonia (UIP) pathology confirmed by lung biopsy. Cases were reviewed with expertise from radiologists, pathologists and clinicians at the local enrolling center. The number of biopsy proven cases was 35 of 60 patients, representing 58% of the study cohort. All cases and controls were of Caucasian ethnicity.

### Sample acquisition and preparation

Peripheral blood was collected in EDTA-containing vacutainers at study centers and samples were shipped by overnight mail using cold packs to the University of Michigan. Samples were collected at 3 time points, namely screening, week 48 and week 80. Samples from healthy human controls were obtained from MedImmune and analyzed simultaneously with the COMET specimens. Whole blood was centrifuged at 2500 rpm for 10 minutes and plasma was collected and frozen at −80 °C in small aliquots. Samples were shipped to SomaLogics for analysis on the SOMAscan® panel (1129 analytes). Plasma samples were diluted at 3 different concentrations for analysis on the aptamer array at the optimal concentrations for each SOMAmer^©^.

### SOMAscan Assay

The SOMAscan® proteomic assay has been described extensively in previous publications^25^. In brief, each of the listed proteins is measured using a modified aptamer reagent and measured quantitatively in relative fluorescence units (RFU’s) using a custom Agilent hybridization chip. Normalization and inter-run calibration were performed according to SOMAscan v3 assay data quality-control procedures as defined in the SomaLogic good laboratory practice quality system. A complete list of SOMAscan^©^ analytes may be found online (http://www.somalogic.com/somalogic/media/Assets/PDFs/SSM-045-REV-1-SOMAscan-Assay-1-3k-Content.pdf).

### Statistical Analysis of SOMAscan assay results

Proteomic data is reported quantitatively as RFU’s for 1129 analytes in 60 IPF patients and 21 healthy controls. For a graphic summary of our investigative approach see Supplemental Figure S1.

The initial approach first identified 203 proteins that differentiated IPF from controls. Relative fold change in blood protein levels were calculated by dividing the average intensity in IPF samples by the average intensity in the healthy samples. Statistical analysis between the healthy and IPF patients was performed by a standard two-tailed and two-sample t-test. Graphical representation of the proteomic data was created using GraphPad Prism software (v6.01 for Windows, GraphPad Software, La Jolla, CA). Significantly different proteins were those that passed a set false discovery rate threshold of 1%. Hierarchical clustering of significantly different proteins was generated by unsupervised average linkage hierarchical clustering using Pearson’s correlation coefficient as the distance metric^56^.

Upon comparison of epidemiological factors between the two groups, we found age to be slightly increased in the normal group. To account for this and identify age-adjusted proteomic differences, we performed linear regression with all biomarkers and age as predictors based on comparison between the IPF and normal cohort, and assessed mean analyte differences between IPF patients and controls adjusted for age. To account for multiple comparisons, we considered Benjamini-Hochberg false discovery rate methods^57^, but eventually decided upon a more conservative Bonferroni correction to maintain an overall type I error of 0.01 and more aggressively screen analytes from the pool of candidates^58^,^59^. Altogether, this resulted in a refined volcano plot showing the age-adjusted proteome. Hierarchical clustering was then used to visualize how these proteins differentiated the healthy and IPF patients.

### Analysis of the differentially expressed IPF proteome with DAVID and Cytoscape

To identify significantly enriched biological process that differentiate IPF from control, those proteins that passed initial screening steps (a Bonferroni correction and linear regression modelling for age) were catalogued into “upregulated” and “downregulated” profiles. In brief, proteins that were meaningfully “upregulated” or “downregulated” were deemed to have potentially significant biological roles in IPF patients compared to the control cohort. A fold increase over control mean of 1.5 and a fold decrease below control mean of 0.75 were used as thresholds for “upregulated” and “downregulated” proteins, respectively. These criteria selected out 48 upregulated proteins and 116 downregulated proteins when comparing IPF patients to controls (Supplementary Tables S2 and S3). Certain proteins were measured in combination (see Supplementary Tables S2 and S3). Certain proteins, i.e. inactivated or splice variants, measured by the SOMASCAN array do not have unique UniProt identifiers available, and therefore the parent protein UniProt Identifier is reported. Functional annotation and visualization was employed using the Cytoscape (v3.3.0) software environment and the ClueGO (v2.2.5) plugin application^60^,^61^. In brief, for ClueGo analysis, Gene ontology levels and Kegg Pathways were explored with medium specificity and a Kappa score of >0.4. The Bonferroni correction was employed for each P value calculation. GO fusion was used to reduce redundancy with child-parent term fusion. P value of 0.05 was regarded as significant. Visualization was applied with Overview term labelling and term P value for nodal size. Functional annotation clustering and enrichment analysis was performed using Gene Ontology (GO) biological processes (BP FAT), molecular function (MF FAT), Kyoto Encyclopedia of Genes and Genomes (KEGG). Enrichment analysis was undertaken by submitting these proteins to the Database for Annotation, Visualization and Integrated Discovery (DAVID) (http://david.abcc.ncifcrf.gov/)^62^,^63^. Enrichment analysis was performed on the basis of *uniprot_accession* as identifier and *gene list* as list type, medium stringency and Bonferroni correction was applied. Enrichment chart analysis was performed using Gene Ontology (GO) biological processes (BP FAT), GO molecular function (MF FAT) and Kyoto Encyclopedia of Genes and Genomes (KEGG). The top functional annotation clusters with significant enrichment scores were identified.

### Identification of a minimal IPF proteomic signature with hierarchical clustering and PLSDA

The Least Absolute Shrinkage and Selection Operator (LASSO) method^56^ was used to identify a minimum, age-adjusted protein signature that best differentiated IPF and normal proteomes and was implemented using Matlab software^64^ (Mathworks, Natick, MA). *K*-fold cross-validation was used to generate the model that had the lowest possible mean squared error for prediction. Associated features for this model were chosen as the minimum set of biomarkers. In order to allow for age-adjustment in the LASSO model, age was forced into the model as a parameter and assigned zero penalty. PLSDA assessed the usefulness of the LASSO-identified protein signature for differentiating healthy and IPF patients. Data were normalized with mean centering and variance scaling, and cross-validation was performed by iteratively excluding random subsets in groups of 9-10 data points during model calibration. Excluded data samples would then be used to test model predictions. Hierarchical clustering of LASSO-identified proteins was generated by unsupervised average linkage hierarchical clustering using Pearson’s correlation coefficient as the distance metric.

### Investigating the Effect of Comorbidities in IPF had on the LASSO and PLSDA Analysis

To investigate whether or not the comorbidities present in some IPF patients affected the feature selection by LASSO or the clustering in PLSDA, we performed a principal component analysis (PCA) on all of the measured blood proteins in the healthy and IPF patients. PCA was chosen as the method of analysis due to the lack of knowledge of the comorbidities seen within the healthy cohort. Gastroesophageal reflux disease (GERD) and obstructive sleep apnea (OSA) were examined based on their prevalence in the IPF patients (34 patients with GERD and 12 patients with OSA).

## Additional Information

**How to cite this article:** O’Dwyer, D. N. *et al*. The peripheral blood proteome signature of idiopathic pulmonary fibrosis is distinct from normal and is associated with novel immunological processes. *Sci. Rep.* **7**, 46560; doi: 10.1038/srep46560 (2017).

**Publisher's note:** Springer Nature remains neutral with regard to jurisdictional claims in published maps and institutional affiliations.

## Supplementary Material

Supplementary Information
