# Erratum: The peripheral blood proteome signature of idiopathic pulmonary fibrosis is distinct from normal and is associated with novel immunological processes

**DOI:** 10.1038/srep46860

**Published:** 2017-06-30

**Authors:** David N. O’Dwyer, Katy C. Norman, Meng Xia, Yong Huang, Stephen J. Gurczynski, Shanna L. Ashley, Eric S. White, Kevin R. Flaherty, Fernando J. Martinez, Susan Murray, Imre Noth, Kelly B. Arnold, Bethany B. Moore

Scientific Reports 7: Article number: 4656010.1038/srep46560; published online: 04 25 2016; updated: 06 30 2017

The original version of this Article contained an error in the reference list, where reference 51 was incorrectly listed as “Ogawa, S. *et al. Reduced cerebrospinal fluid ethanolamine concentration in major depressive disorder*. *Sci Rep* **5**, 7796, doi: 10.1038/srep07796 (2015).” The correct reference 51 appears below.

This has now been corrected in the HTML and PDF versions of this Article.
