# Supplementary material for: The peripheral blood proteome signature of idiopathic pulmonary fibrosis is distinct from normal and is associated with novel immunological processes
Source: Sci Rep. 2017 Apr 25;7:46560. doi: 10.1038/srep46560 (PMC5404506; doi:10.1038/srep46560)

The peripheral blood proteome signature of idiopathic pulmonary fibrosis is distinct from normal and is associated with novel immunological processes

*David N. O'Dwyer[1†], Katy C. Norman[2†], Meng Xia[3], Yong Huang[4] ,Stephen J. Gurczynski[1], Shanna L. Ashley[5] , Eric S. White[1], Kevin R. Flaherty[1], Fernando J. Martinez[6], Susan Murray[3], Imre Noth[4], Kelly B. Arnold[2,#] and Bethany B. Moore[1,7,#]

Table S1 Study population demographics

	IPF	Normal controls	P value*
Mean age in yrs. (SD)	64.56 (7.74)	69.29 (10.12)	0.0257
Male No. (%)	41 (68.33)	15 (71.43)	0.7916
Smoking status			
Never	19 (31.66)	N/A	_
Ex	40 (66.66)	N/A	_
Current	1 (1.66)	N/A	_

*Students t-test and Pearson χ2 squared test respectively. SD: standard deviation

Table S2 **List of relevant co-morbidities and their frequencies with the COMET IPF patient cohort**

Co-morbidity	Freq (N=60) (%)
CAD	
Yes	7 (11.67)
No	53 (88.33)
MI	
Yes	2 (3.33)
No	58 (96.67)
Lung Cancer	
Yes	1 (1.67)
No	59 (98.33)
Other Cancer	
Yes	5 (8.33)
No	55 (91.67)
GERD	
Yes	34 (56.67)
No	25 (41.67)
Unknown	1 (1.67)
OSA	
Yes	12 (20.00)
No	48 (80.00)
Pulm HTN	
Yes	4 (6.67)
No	55 (91.67)
Unknown	1 (1.67)
Emphysema/Bronchitis	
Yes	1 (1.67)
No	58 (96.67)
Unknown	1 (1.67)

CAD-coronary artery disease: MI – myocardial infarction: GERD – gastroesophageal reflux disease: OSA – obstructive sleep apnea: Pulm HTN – pulmonary hypertension.

Table S3 **List of upregulated proteins in the IPF peripheral proteome compared to control**

Protein	UniProt ID	Gene ID
Afamin	P43652	AFM
Aflatoxin B1 aldehyde reductase member 2	O43488	AKR7A2
AH receptor-interacting protein	O00170	AIP
Alpha-soluble NSF attachment protein	P54920	NAPA
Aminoacylase-1	Q03154	ACY1
Apolipoprotein A-I	P02647	APOA1
Beta-Ala-His dipeptidase	Q96KN2	CNDP1
Bone morphogenetic protein 1	P13497	BMP1
C5a anaphylatoxin	P01031	C5
Cathepsin B	P07858	CTSB
cGMP-specific 3',5'-cyclic phosphodiesterase	O76074	PDE5A
Chloride intracellular channel protein 1	O00299	CLIC1
Coagulation Factor V	P12259	F5
Complement C1r subcomponent	P00736	C1R
Complement C4	P0C0L4	C4A
Cyclin-dependent kinase 8:Cyclin-C complex	P49336, P24863	CDK8 CCNC
Dual 3',5'-cyclic-AMP and -GMP phosphodiesterase 11A	Q9HCR9	PDE11A
Dual specificity mitogen-activated protein kinase kinase 4	P45985	MAP2K4
Endothelin-converting enzyme 1	P42892	ECE1
Fibronectin	P02751	FN1
Glyceraldehyde-3-phosphate dehydrogenase	P04406	GAPDH
Glycogen synthase kinase-3 alpha/beta	P49840, P49841	GSK3A GSK3B
Growth hormone receptor	P10912	GHR
Growth/differentiation factor 11	O95390	GDF11
GTP-binding nuclear protein Ran	P62826	RAN
Intercellular adhesion molecule 5	Q9UMF0	ICAM5
MAP kinase-activated protein kinase 2	P49137	MAPKAPK2

Matrilysin	P09237	MMP7
Methionine aminopeptidase 2	P50579	METAP2
Nascent polypeptide-associated complex subunit alpha	Q13765	NACA
Peptidyl-prolyl cis-trans isomerase D	Q08752	PPID
Phosphatidylinositol 4,5-bisphosphate 3-kinase catalytic subunit alpha isoform: Phosphatidylinositol 3-kinase regulatory subunit alpha complex	P42336, P27986	PIK3CA PIK3R1
Plasma serine protease inhibitor	P05154	SERPINA5
Proprotein convertase subtilisin/kexin type 7	Q16549	PCSK7
Protein kinase C alpha type	P17252	PRKCA
Protein kinase C beta type (splice variant beta-II)	P05771	PRKCB
Proto-oncogene tyrosine-protein kinase Src	P12931	SRC
P-Selectin	P16109	SELP
Pyruvate kinase PKM	P14618	PKM2
Ras-related C3 botulinum toxin substrate 1	P63000	RAC1
Ribosome maturation protein SBDS	Q9Y3A5	SBDS
Small glutamine-rich tetratricopeptide repeat-containing protein alpha	O43765	SGTA
Sorting nexin-4	O95219	SNX4
Sphingosine kinase 1	Q9NYA1	SPHK1
Tumor necrosis factor ligand superfamily member 13B	Q9Y275	TNFSF13B
Tyrosine-protein kinase BTK	Q06187	BTK
Tyrosine-protein kinase CSK	P41240	CSK
Tyrosine-protein kinase Tec	P42680	TEC

Uniprot Accession ID listed. Detailed descriptions of proteins available at http://www.uniprot.org/
N=48 proteins
*Measured in combination

Table S4 List of downregulated proteins in the IPF peripheral proteome compared to control

Protein	UniProt ID	Gene ID
Allograft inflammatory factor 1	P55008	AIF1
Alcohol dehydrogenase [NADP(+)]	P14550	AKR1A1
Alkaline phosphatase, tissue-nonspecific isozyme	P05186	ALPL
Annexin A1	P04083	ANXA1
Annexin A2	P07355	ANXA2
Complement C3	P01024	C3
Complement C3b, inactivated	P01024	C3
Complement C4b	P0C0L5	C4B
Carbonic anhydrase 3	P07451	CA3
Calcium/calmodulin-dependent protein kinase type II subunit beta	Q13554	CAMK2B
Calcium/calmodulin-dependent protein kinase type II subunit delta	Q13557	CAMK2D
Macrophage-capping protein	P40121	CAPG
Caspase-10	Q92851	CASP10
Calpastatin	P20810	CAST
C-C motif chemokine 14	Q16627	CCL14
C-C motif chemokine 23	P55773	CCL23
Cyclin-dependent kinase inhibitor 1B	P46527	CDKN1B
Cryptic protein	P0CG37	CFC1
Cofilin-1	P23528	CFL1
Chymase	P23946	CMA1
C-reactive protein	P02741	CRP
Macrophage colony-stimulating factor 1	P09603	CSF1
Granulocyte-macrophage colony-stimulating factor	P04141	CSF2
Cystatin-C	P01034	CST3
Cathepsin S	P25774	CTSS
C-X-C motif chemokine 11	O14625	CXCL11
Interleukin-8	P10145	CXCL8

Protein	UniProt ID	Gene ID
Discoidin domain-containing receptor 2	Q16832	DDR2
Eukaryotic translation initiation factor 4 gamma 2	P78344	EIF4G2
Eukaryotic translation initiation factor 5	P55010	EIF5
Eukaryotic translation initiation factor 5A-1	P63241	EIF5A
Ephrin type-A receptor 2	P29317	EPHA2
Tissue Factor	P13726	F3
Ficolin-1	O00602	FCN1
Tyrosine-protein kinase Fyn	P06241	FYN
Growth/differentiation factor 5	P43026	GDF5
Aspartate aminotransferase, cytoplasmic	P17174	GOT1
Glucose-6-phosphate isomerase	P06744	GPI
Glutathione S-transferase P	P09211	GSTP1
Histone H2A.z	P0C0S5	H2AFZ
Hepatitis A virus cellular receptor 2	Q8TDQ0	HAVCR2
Hepatoma-derived growth factor-related protein 2	Q7Z4V5	HDGFRP2
Histone H1.2	P16403	HIST1H1C
High mobility group protein B1	P09429	HMGB1
Heme oxygenase 2	P30519	HMOX2
Heterogeneous nuclear ribonucleoproteins A2/B1	P22626	HNRNPA2B1
Heterogeneous nuclear ribonucleoprotein A/B	Q99729	HNRNPAB
Estradiol 17-beta-dehydrogenase 1	P14061	HSD17B1
Heat shock 70 kDa protein 1A/1B	P08107	HSPA1A
Serine protease HTRA2, mitochondrial	O43464	HTRA2
ICOS ligand	O75144	ICOSLG
Insulin-like growth factor-binding protein 1	P08833	IGFBP1
Insulin-like growth factor-binding protein 2	P18065	IGFBP2
Interleukin-16	Q14005	IL16
Interleukin-2	P60568	IL2

Protein	UniProt ID	Gene ID
Interleukin-3	P08700	IL3
Integrin alpha-I: beta-1 complex	P56199, P05556	ITGA1 ITGB1
Killer cell immunoglobulin-like receptor 2DL4	Q99706	KIR2DL4
Importin subunit alpha-1	P52292	KPNA2
Lipopolysaccharide-binding protein	P18428	LBP
Neutrophil gelatinase-associated lipocalin	P80188	LCN2
Lactotransferrin	P02788	LTF
Dual specificity mitogen-activated protein kinase kinase 1	Q02750	MAP2K1
Dual specificity mitogen-activated protein kinase kinase 2	P36507	MAP2K2
Mitogen-activated protein kinase 13	O15264	MAPK13
Myoglobin	P02144	MB
Matrix metalloproteinase-9	P14780	MMP9
Myeloperoxidase	P05164	MPO
Moesin	P26038	MSN
Nicotinamide phosphoribosyltransferase	P43490	NAMPT
NudC domain-containing protein 3	Q8IVD9	NUDCD3
Oxidized low-density lipoprotein receptor 1	P78380	OLR1
Protein DJ-1	Q99497	PARK7
Phosphatidylethanolamine-binding protein 1	P30086	PEBP1
Prefoldin subunit 5	Q99471	PFDN5
Phosphoglycerate mutase 1	P18669	PGAM1
Peptidoglycan recognition protein 1	O75594	PGLYRP1
Elafin	P19957	PI3
Phospholipase A2, membrane associated	P14555	PLA2G2A
Urokinase plasminogen activator surface receptor	Q03405	PLAUR
NADPH--cytochrome P450 reductase	P16435	POR
Myeloblastin	P24158	PRTN3
Proteasome subunit alpha type-2	P25787	PSMA2

Protein	UniProt ID	Gene ID
Prostaglandin G/H synthase 2	P35354	PTGS2
Tyrosine-protein phosphatase non-receptor type 1	P18031	PTPN1
Tyrosine-protein phosphatase non-receptor type 11	Q06124	PTPN11
Tyrosine-protein phosphatase non-receptor type 6	P29350	PTPN6
RNA-binding protein 39	Q14498	RBM39
Resistin	Q9HD89	RETN
Ubiquitin	P62979	RPS27A
Ubiquitin+1, truncated mutation for UbB	P62979	RPS27A
40S ribosomal protein S7	P62081	RPS7
Protein S100-A9	P06702	S100A9
Serum amyloid A-1 protein	P0DJI8	SAA1
Scavenger receptor class F member 1	Q14162	SCARF1
alpha-1-antichymotrypsin complex	P01011	SERPINA3
Plasma protease C1 inhibitor	P05155	SERPING1
Pulmonary surfactant-associated protein D	P35247	SFTPD
SHC-transforming protein 1	P29353	SHC1
Sialic acid-binding Ig-like lectin 14	Q08ET2	SIGLEC14
Small nuclear ribonucleoprotein F	P62306	SNRPF
FACT complex subunit SSRP1	Q08945	SSRP1
Heterogeneous nuclear ribonucleoprotein Q	O60506	SYNCRIP
Trefoil factor 3	Q07654	TFF3
Metalloproteinase inhibitor 1	P01033	TIMP1
Tumor necrosis factor receptor superfamily member 1B	P20333	TNFRSF1B
Tumor necrosis factor ligand superfamily member 14	O43557	TNFSF14
DNA topoisomerase 1	P11387	TOP1
Triosephosphate isomerase	P60174	TPI1
SUMO-conjugating enzyme UBC9	P63279	UBE2I
Ubiquitin-conjugating enzyme E2 N	P61088	UBE2N

Protein	UniProt ID	Gene ID
Ubiquitin-fold modifier 1	P61960	UFM1
Vacuolar protein sorting-associated protein VTA1 homolog	Q9NP79	VTA1
X-ray repair cross-complementing protein 6	P12956	XRCC6
Tyrosine-protein kinase Yes	P07947	YES1

* P08107 updated as secondary accession to P0DMV8/P0DMV9 (HSPA1A/HSPA1B).
N= 116

Table S5 List of all significant proteins on analysis of IPF proteome versus healthy

Uniprot ID	Gene ID	Fold expression	Predicted Linear Model Ratios (IPF/Healthy)	P-values significant after Bonferroni correction	FDR	Age-adjusted P-values significant after Bonferroni Correction	Age-adjusted FDR
P17252	PRKCA	2.844150745	2.7443155	1.36E-14	0.000194363	4.3293E-14	0.001000972
P02647	APOA1	1.542341061	1.544871222	1.451E-06	0.001467444	4.2806E-09	0.001282799
O95390	GDF11	1.628565596	1.608156622	7.663E-12	0.000359572	1.1845E-07	0.000855199
P09237	MMP7	2.654602809	2.747872619	2.054E-18	0.000126336	2.5766E-10	0.001720117
P01031	C5	1.654531862	1.675076486	5.828E-07	0.001282799	7.3906E-06	0.001681244
P63000	RAC1	1.610302077	1.574044262	1.098E-06	0.001389699	6.2295E-06	0.00191448
Q9Y275	TNFSF13B	2.129450761	2.191299147	3.817E-19	9.71817E-05	4.4978E-11	0.000272109
P07858	CTSB	1.665079978	1.742647874	3.293E-13	0.000242954	3.113E-11	0.000437318
P50579	METAP2	2.400193949	2.369123805	4.876E-10	0.000515063	2.655E-08	0.002147716
P00736	C1R	4.387258094	4.463494883	7.042E-22	5.8309E-05	6.7295E-17	0.001574344
P13497	BMP1	2.462088383	2.418442708	4.891E-16	0.000165209	4.7633E-14	0.002196307
P49336, P24863	CDK8 CCNC	1.642587894	1.64618749	5.658E-19	0.000116618	8.7935E-11	0.00292517
P41240	CSK	1.955228256	1.939577175	5.987E-10	0.000524781	2.9947E-06	0.001137026
P05154	SERPINA5	2.493816792	2.438316738	4.487E-18	0.000136054	2.0621E-20	0.001525753
P49840, P49841	GSK3A GSK3B	3.734712707	3.634606001	3.89E-27	9.71817E-06	6.23E-18	0.001389699
P42680	TEC	2.558122735	2.478830736	2.333E-16	0.000145773	2.3177E-08	0.001156463
P49137	MAPKAPK2	1.868501241	1.883819574	4.897E-08	0.00090379	1.7658E-06	0.002400389
O00170	AIP	1.715314482	1.675073096	9.679E-15	0.000174927	6.676E-09	0.000719145
P04406	GAPDH	1.669919932	1.639169965	5.773E-06	0.001856171	5.3425E-07	0.000942663
Q13765	NACA	2.276481926	2.165538354	3.917E-21	7.77454E-05	6.6077E-12	0.00047619
O43765	SGTA	1.859855557	1.803317323	1.199E-08	0.000767736	2.8969E-07	0.002001944
O95219	SNX4	2.060637667	1.939104225	7.436E-09	0.000709427	1.9616E-08	0.002137998
P02751	FN1	2.058884999	2.1387836	5.309E-12	0.000340136	2.7176E-08	0.001059281
O43488	AKR7A2	2.382612119	2.339107573	2.438E-11	0.000388727	1.7054E-07	0.00159378
P14618	PKM2	2.372734125	2.397160175	2.892E-13	0.000233236	5.4956E-09	0.002254616
P62826	RAN	10.78442484	8.501570061	9.529E-21	8.74636E-05	3.8826E-11	0.001467444
P54920	NAPA	2.259133786	2.144386113	1.622E-13	0.000204082	1.6544E-09	0.002983479
Q9NYA1	SPHK1	4.924705886	4.492447031	2.931E-21	6.80272E-05	1.0608E-13	0.000359572

Uniprot ID	Gene ID	Fold expression	Predicted Linear Model Ratios (IPF/Healthy)	P-values significant after Bonferroni correction	FDR	Age-adjusted P-values significant after Bonferroni Correction	Age-adjusted FDR
Q16549	PCSK7	2.06801082	1.986426465	5.236E-22	4.85909E-05	6.9419E-12	0.000291545
P12259	F5	1.568462646	1.611038214	1.518E-11	0.000369291	1.0352E-06	0.000349854
O00299	CLIC1	3.059946004	2.993311375	6.309E-12	0.000349854	6.6552E-09	2.91545E-05
Q9UMF0	ICAM5	1.759344079	1.804719167	2.014E-11	0.000379009	1.3082E-07	0.000281827
Q08752	PPID	3.742581674	3.511759899	1.307E-12	0.000281827	3.2523E-06	0.0003207
P45985	MAP2K4	1.747910459	1.715622054	4.992E-09	0.000680272	9.5677E-08	0.000330418
P12931	SRC	3.854282773	3.709558785	1.198E-22	3.88727E-05	1.027E-18	0.000252672
O76074	PDE5A	4.43765169	3.978480542	9.751E-26	1.94363E-05	8.5254E-15	0.000447036
P10912	GHR	1.777326642	1.767808273	1.284E-10	0.000447036	2.1388E-10	0.000728863
Q03154	ACY1	4.904977655	4.13058665	6.462E-13	0.000272109	4.2266E-06	0.000155491
P42336, P27986	PIK3CA PIK3R1	1.595594423	1.612386386	5.423E-19	0.0001069	6.2975E-11	0.001972789
Q06187	BTK	10.44813268	9.363373991	2.252E-24	2.91545E-05	8.0073E-14	0.001068999
Q96KN2	CNDP1	2.772175242	2.868771762	5.328E-13	0.000262391	5.454E-10	0.001127308
Q9Y3A5	SBDS	2.559809181	2.525581685	1.145E-14	0.000184645	8.1077E-10	0.000612245
P16109	SELP	1.755253189	1.688921305	5.013E-11	0.000398445	4.4751E-09	0.001292517
P08649	C4A C4B	2.163849532	2.152620093	2.106E-13	0.000223518	1.7857E-12	0.000894072
P43652	AFM	1.516790465	1.509684337	4.459E-09	0.000660836	1.8907E-10	0.001613217
Q9HCR9	PDE11A	2.693926113	2.629765299	4.46E-10	0.000505345	4.4336E-07	0.000262391
P05771	PRKCB	2.549572043	2.527742077	2.072E-13	0.0002138	1.944E-07	0.000621963
P42892	ECE1	1.496922719	1.545032257	2.628E-08	0.000864917	1.3217E-07	0.002439261
Q9UHD0	IL19	1.536009374	1.522027626	6.889E-08	0.000942663	2.5591E-05	0.001146744
P23280	CA6	2.120863591	2.199196968	8.504E-07	0.001350826	9.6872E-05	0.000242954
Q8N1Q1	CA13	2.14243776	2.090315543	3.449E-08	0.000874636	0.00011986	0.001428571
P07996	THBS1	1.722022566	1.66082076	2.042E-06	0.001564626	0.00124037	0.002954325
Q9NQU5	PAK6	1.78765298	1.782944743	9.87E-07	0.001379981	3.1333E-05	0.001049563
Q8N5S9	CAMKK1	1.689904266	1.68676957	9.496E-07	0.001370262	0.00133575	0.001477162
Q99714	HSD17B10	2.648891087	2.544580128	1.222E-07	0.001039845	0.00027562	0.001234208
Q08209, P63098	PPP3CA PPP3R1	1.996632503	2.050362653	2.685E-07	0.001175899	3.7241E-05	3.88727E-05
P03956	MMP1	1.880033667	1.868914014	6.063E-08	0.000932945	9.6125E-05	0.001448008
P15514	AREG	1.597172106	1.537410827	1.067E-07	0.000991254	6.0986E-05	0.000699708

Uniprot ID	Gene ID	Fold expression	Predicted Linear Model Ratios (IPF/Healthy)	P-values significant after Bonferroni correction	FDR	Age-adjusted P-values significant after Bonferroni Correction	Age-adjusted FDR
P36888	FLT3	1.633132296	1.62729439	1.988E-07	0.00111759	0.00026356	0.000602527
P12277, P06732	CKB CKM	3.424539483	2.772971246	9.364E-09	0.000748299	0.00031418	0.001341108
O75636	FCN3	1.486624762	1.505689722	4.105E-06	0.001739553	0.00219711	0.00877551
O43557	TNFSF14	0.437387768	0.433556681	2.631E-09	0.000612245	3.9442E-15	0.001098154
P62306	SNRPF	0.589209938	0.585044542	3.073E-09	0.000631681	2.0066E-16	0.001671526
P18065	IGFBP2	0.561250316	0.560919529	1.127E-06	0.001399417	8.7262E-10	0.000641399
P14780	MMP9	0.580646533	0.581799239	8.175E-06	0.001963071	6.1719E-07	0.000340136
P05164	MPO	0.66653812	0.664052177	3.288E-07	0.001195335	3.5821E-06	0.000116618
P55010	EIF5	0.264516494	0.257394258	1.575E-10	0.000466472	2.9209E-26	0.00068999
P30519	HMOX2	0.302673799	0.309421198	1.115E-08	0.000758017	2.4311E-15	0.00079689
Q03405	PLAUR	0.714503307	0.711668768	3.972E-06	0.001729835	6.9874E-07	0.000553936
P01024	C3	0.531153018	0.521491138	3.962E-12	0.0003207	6.326E-28	0.000631681
P16435	POR	0.193507136	0.203838807	1.288E-07	0.001049563	1.2994E-16	0.001554908
P43026	GDF5	0.527925041	0.526377553	1.399E-07	0.001098154	5.7904E-16	0.000369291
P01024	C3	0.594138865	0.588274349	2.357E-08	0.000855199	7.0592E-16	0.000631681
Q14005	IL16	0.23033519	0.237400985	4.271E-09	0.000651118	2.2301E-17	0.000660836
P12956	XRCC6	0.179825644	0.192845513	9.608E-08	0.000971817	4.8595E-14	0.001758989
P55008	AIF1	0.344025162	0.34967201	5.447E-08	0.000913508	7.8303E-12	6.80272E-05
P52292	KPNA2	0.576832085	0.585079807	5.707E-09	0.00068999	1.2065E-17	0.001166181
Q02750	MAP2K1	0.359077237	0.362158859	4.702E-07	0.001234208	8.6888E-13	8.74636E-05
P11387	TOP1	0.161052587	0.172640307	7.554E-06	0.001943635	2.8135E-13	0.000544218
P63279	UBE2I	0.402202186	0.406545614	1.118E-07	0.001000972	9.1848E-12	0.000913508
P07947	YES1	0.530683894	0.535492527	5.545E-08	0.000923226	1.0937E-15	0.000515063
P16403	HIST1H1C	0.18202697	0.179435862	2.033E-12	0.000301263	1.1917E-21	0.000991254
P18031	PTPN1	0.513454609	0.518122474	4.335E-06	0.001749271	2.7091E-11	0.000145773
P20810	CAST	0.625591416	0.647812276	1.392E-10	0.000456754	1.1152E-10	0.000466472
P02144	MB	0.444987418	0.457379403	1.781E-06	0.001516035	1.3409E-12	0.000680272
P60568	IL2	0.602649926	0.607874718	8.102E-07	0.001341108	8.832E-12	7.77454E-05
P25774	CTSS	0.691366271	0.70449585	1.438E-06	0.001457726	2.5524E-06	1.94363E-05
P0CG37	CFC1	0.480568112	0.480547449	1.329E-07	0.001078717	6.6844E-15	9.71817E-05

Uniprot ID	Gene ID	Fold expression	Predicted Linear Model Ratios (IPF/Healthy)	P-values significant after Bonferroni correction	FDR	Age-adjusted P-values significant after Bonferroni Correction	Age-adjusted FDR
O43464	HTRA2	0.727295489	0.719331748	7.34E-07	0.001321672	2.8688E-09	0.000485909
O75594	PGLYRP1	0.385704603	0.406150492	1.661E-06	0.001506317	1.0747E-11	0.000126336
Q13554	CAMK2B	0.448493259	0.46201085	3.863E-06	0.001720117	8.3106E-06	0.000651118
Q06124	PTPN11	0.325170474	0.320946853	7.107E-11	0.000417881	7.5569E-24	0.00133139
Q13557	CAMK2D	0.448876214	0.457971263	1.144E-06	0.001409135	3.2017E-08	0.000923226
P10145	CXCL8	0.034225488	0.036256866	3.956E-08	0.000894072	7.9872E-23	0.000194363
P56199, P05556	ITGA1 ITGB1	0.298316266	0.315384647	5.57E-07	0.001263362	1.0694E-12	5.8309E-05
P24158	PRTN3	0.241989732	0.250856356	2.183E-06	0.001584062	1.1768E-09	0.00058309
Q16832	DDR2	0.59816339	0.593148426	1.398E-06	0.001448008	8.3643E-13	0.001088435
O00602	FCN1	0.487691807	0.491252641	4.684E-06	0.001788144	2.947E-09	0.000388727
P36507	MAP2K2	0.399325508	0.409209093	3.71E-09	0.000641399	1.0395E-20	0.000301263
P46527	CDKN1B	0.390461304	0.389275226	1.256E-09	0.000592809	8.6472E-14	0.001205053
P09603	CSF1	0.349185705	0.345595517	5.652E-06	0.001846453	2.4947E-11	0.001253644
P35354	PTGS2	0.300945517	0.304112877	8.668E-10	0.000563654	3.2731E-23	4.85909E-05
P06241	FYN	0.15968612	0.158580485	9.413E-11	0.0004276	3.0673E-28	0.001039845
P62081	RPS7	0.185649796	0.187803316	6.968E-10	0.000553936	4.3763E-22	0.0005345
P18669	PGAM1	0.380200082	0.367406052	2.737E-10	0.000485909	2.1317E-13	9.71817E-06
P08107	HSPA1A	0.329230499	0.325625349	3.616E-13	0.000252672	5.5044E-21	0.000184645
P01011	SERPINA3	0.457669471	0.44651438	5.712E-07	0.001273081	9.634E-12	0.000670554
P14550	AKR1A1	0.591448395	0.596031761	3.696E-06	0.001690962	1.6211E-07	0.000505345
P23528	CFL1	0.669498441	0.658275648	7.5E-06	0.001933916	1.3489E-09	0.000204082
O60506	SYNCRIP	0.291191938	0.297780019	9.354E-08	0.000962099	4.8968E-20	0.0001069
Q99471	PFDN5	0.254728054	0.25020284	6.318E-09	0.000699708	1.7807E-24	0.000563654
P06744	GPI	0.529843615	0.523601892	1.48E-08	0.00079689	2.6058E-12	0.001933916
P30086	PEBP1	0.628052496	0.616272435	4.374E-06	0.001758989	1.2629E-08	0.000738581
Q14498	RBM39	0.17003682	0.17844853	1.313E-07	0.001059281	1.2501E-14	0.000592809
P29350	PTPN6	0.339639104	0.338090817	9.057E-10	0.000573372	2.0333E-16	0.000408163
P02741	CRP	0.625041071	0.602656298	1.204E-06	0.001418853	5.8331E-08	0.000233236
Q9UIK4	DAPK2	0.400809219	0.401459963	2.019E-08	0.000826045	1.0825E-07	0.000952381
P35247	SFTPD	0.226428987	0.220331959	4.803E-12	0.000330418	1.8116E-25	0.000767736

Uniprot ID	Gene ID	Fold expression	Predicted Linear Model Ratios (IPF/Healthy)	P-values significant after Bonferroni correction	FDR	Age-adjusted P-values significant after Bonferroni Correction	Age-adjusted FDR
Q99729	HNRNPAB	0.345921247	0.35846041	1.945E-06	0.001554908	4.6961E-09	0.001953353
P62979	RPS27A	0.470375766	0.461535957	5.172E-07	0.001243926	9.9226E-13	0.0002138
Q7Z4V5	HDGFRP2	0.060924947	0.055653979	6.293E-11	0.000408163	4.0153E-34	0.001243926
P14061	HSD17B1	0.176376578	0.186493411	1.353E-07	0.001088435	4.8554E-20	0.002410107
P08700	IL3	0.62221433	0.627986419	3.837E-06	0.001710398	1.9802E-11	0.000223518
P09211	GSTP1	0.544764141	0.534249412	3.899E-07	0.001205053	3.0047E-13	0.000165209
P17174	GOT1	0.524455222	0.517574344	1.944E-09	0.000602527	5.2867E-19	0.000310982
P07355	ANXA2	0.298075338	0.296106201	3.332E-10	0.000495627	4.6371E-22	0.001788144
P40121	CAPG	0.231802025	0.233433389	1.208E-09	0.00058309	1.4771E-12	0.00244898
O15264	MAPK13	0.299068474	0.301080845	1.696E-08	0.000806608	5.1201E-11	0.001875607
P26038	MSN	0.351191486	0.349889679	2.743E-09	0.000621963	3.0007E-12	0.002478134
P43490	NAMPT	0.240390948	0.256498616	3.815E-08	0.000884354	2.7028E-14	0.001584062
Q99497	PARK7	0.310701688	0.322106754	1.307E-08	0.000777454	1.2199E-18	0.00324587
O75144	ICOSLG	0.219547354	0.223111787	1.566E-07	0.001107872	2.0022E-09	0.00180758
Q99706	KIR2DL4	0.478349129	0.479635576	1.145E-07	0.001030126	7.8372E-11	0.002099125
Q14162	SCARF1	0.516331552	0.508016155	1.754E-08	0.000816327	6.8641E-19	0.003313897
P06702	S100A9	0.591697735	0.586341331	1.214E-06	0.001428571	1.0594E-07	0.001195335
P0C0L5	C4A C4B	0.358507662	0.369051932	1.363E-12	0.000291545	3.8228E-20	0.000524781
P09429	HMGB1	0.379602216	0.375656719	2.211E-08	0.000835763	4.4323E-16	0.000495627
P29353	SHC1	0.39845923	0.390325715	6.392E-10	0.000544218	3.9594E-20	0.00170068
P04083	ANXA1	0.491332759	0.484731275	2.239E-08	0.000845481	7.6468E-10	0.000174927
Q08945	SSRP1	0.064935707	0.074339255	1.314E-07	0.001068999	7.0746E-21	0.00234208
Q92851	CASP10	0.733486915	0.746854294	4.988E-06	0.00180758	1.6671E-06	0.001457726
P22626	HNRNPA2B1	0.304653849	0.304147983	1.089E-10	0.000437318	3.7583E-14	0.001321672
Q05397	PTK2	0.361679962	0.378753188	8.674E-09	0.000738581	2.6631E-05	0.000379009
P01033	TIMP1	0.679269263	0.66674616	0.000391	0.002905734	6.6026E-08	0.008357629
P01034	CST3	0.63498025	0.648738355	0.0021138	0.003488824	3.7008E-06	0.00808552
P02788	LTF	0.646580172	0.660962703	0.0002031	0.002633625	3.8306E-06	0.004723032
P05186	ALPL	0.730903722	0.736706819	7.331E-05	0.002303207	1.5228E-06	0.007609329
P55773	CCL23	0.582518753	0.619691865	0.0005164	0.003061224	3.935E-07	0.003226433

Uniprot ID	Gene ID	Fold expression	Predicted Linear Model Ratios (IPF/Healthy)	P-values significant after Bonferroni correction	FDR	Age-adjusted P-values significant after Bonferroni Correction	Age-adjusted FDR
P07451	CA3	0.595832363	0.560995319	0.0029284	0.003654033	2.8427E-06	0.001350826
P63241	EIF5A	0.639598496	0.626260436	4.906E-05	0.002264334	1.829E-08	0.004752187
P61088	UBE2N	0.679152378	0.668632317	9.424E-05	0.002380952	3.7967E-06	0.002779397
P0C0S5	H2AFZ	0.085008123	0.094716988	1.043E-05	0.002001944	2.6092E-14	0.004344023
Q9NP79	VTA1	0.68463685	0.665210265	1.045E-05	0.002011662	5.6174E-11	0.005344995
P25787	PSMA2	0.390509178	0.357843285	0.0047528	0.003906706	1.1165E-07	0.005004859
P05155	SERPING1	0.407843012	0.377329305	0.002597	0.003586006	1.7868E-08	0.001078717
P04141	CSF2	0.716695601	0.70886671	0.0024597	0.003566569	1.5111E-06	0.005218659
P08833	IGFBP1	0.393702902	0.408439401	0.000134	0.002478134	1.192E-06	0.009339164
P80188	LCN2	0.192644603	0.212966828	0.0001084	0.002419825	2.3708E-10	0.004003887
P62979	RPS27A	0.748787706	0.725982264	0.0001239	0.002458698	1.3893E-10	0.0002138
Q16627	CCL14	0.730237589	0.725946814	0.0004162	0.002944606	7.5672E-06	0.006297376
O14625	CXCL11	0.480301473	0.471102974	0.0003025	0.00281827	1.7786E-07	0.004266278
Q9HD89	RETN	0.53666193	0.538277838	0.0002682	0.002750243	1.1095E-07	0.007832847
P18428	LBP	0.601598528	0.621936449	0.0001787	0.00260447	4.7373E-08	0.010447036
P20333	TNFRSF1B	0.528556645	0.530989059	0.0001396	0.00249757	2.0522E-09	0.004596696
P23946	CMA1	0.422400525	0.449911378	0.000103	0.002410107	6.0735E-08	0.008551992
P78380	OLR1	0.376747719	0.387146365	1.569E-05	0.002060253	4.2928E-09	0.001729835
P61960	UFM1	0.61686735	0.606344498	1.583E-05	0.002069971	1.0983E-09	0.00281827
P78344	EIF4G2	0.517757776	0.509215443	5.586E-05	0.002274052	1.092E-08	0.004013605
Q8IVD9	NUDCD3	0.555472185	0.548221386	1.009E-05	0.001992225	1.1417E-10	0.003119534
P60174	TPI1	0.693640403	0.683253702	8.747E-05	0.002361516	3.7814E-06	0.006287658
P0DJI8	SAA1	0.173531112	0.180295633	0.0004928	0.00303207	3.3565E-08	0.00090379
Q07654	TFF3	0.478602321	0.492382656	0.0051497	0.003974733	8.1428E-06	0.005228377
P13726	F3	0.592836215	0.609717233	0.0001829	0.002614189	2.7012E-07	0.002827988
P19957	PI3	0.359454756	0.367402763	8.107E-05	0.002332362	1.1063E-10	0.00526725
P14555	PLA2G2A	0.164508441	0.178116603	0.0004127	0.002934888	2.7444E-09	0.002322643
P29317	EPHA2	0.543657766	0.539472754	0.0029097	0.003644315	2.3118E-06	0.007152575
Q08ET2	SIGLEC14	0.653055432	0.645056946	0.0003386	0.002857143	8.2826E-06	0.004897959
Q8TDQ0	HAVCR2	0.738663283	0.733196887	0.0002157	0.002691934	8.6442E-06	0.008678328

Uniprot ID	Gene ID	Fold expression	Predicted Linear Model Ratios (IPF/Healthy)	P-values significant after Bonferroni correction	FDR	Age-adjusted P-values significant after Bonferroni Correction	Age-adjusted FDR
P13686	ACP5	1.504767826	1.489334666	4.532E-09	0.000670554	9.3819E-07	0.000777454
P20273	CD22	1.184673275	1.176234949	8.388E-09	0.000728863	3.2038E-06	0.003605442
P51665	PSMD7	1.227734879	1.221843412	6.226E-10	0.0005345	7.0956E-06	0.002050534
Q07817	BCL2L1	1.490277914	1.479585533	2.427E-12	0.000310982	5.7369E-08	0.000884354
P06396	GSN	1.285528406	1.306069247	3.753E-06	0.00170068	7.1603E-07	0.001418853
P04196	HRG	1.418870563	1.442868446	2.642E-07	0.001166181	1.6043E-07	0.001360544
Q9HCK4	ROBO2	1.407713095	1.395479599	1.126E-07	0.00101069	1.6629E-08	0.001516035
P12268	IMPDH2	1.338326362	1.339255747	2.562E-10	0.00047619	5.2757E-06	0.00313897
P22223	CDH3	1.385373136	1.399310188	1.063E-07	0.000981535	2.2775E-07	0.003634597
P07225	PROS1	1.272634163	1.275797707	2.912E-07	0.001185617	4.6845E-09	0.002176871
O43291	SPINT2	1.485466338	1.458185513	1.299E-06	0.00143829	1.8954E-06	0.001982507
Q9BY41	HDAC8	1.424686176	1.425858407	2.406E-16	0.000155491	1.5161E-09	0.006598639
P08697	SERPINF2	1.31047173	1.30667218	1.367E-08	0.000787172	3.6237E-11	0.002934888
P02748	C9	0.781050273	0.782880587	1.145E-07	0.001020408	1.3663E-07	0.002332362
P29622	SERPINA4	1.269072953	1.257079381	8.1E-06	0.001953353	3.5722E-06	0.001496599
Q96IY4	CPB2	0.819875158	0.824769315	4.543E-06	0.001778426	4.0513E-07	0.003449951
P31785	IL2RG	1.545659909	1.496723647	6.757E-07	0.001311953	0.00018474	0.0004276
P26951	IL3RA	1.508301381	1.468159905	2.326E-06	0.00159378	0.00142495	0.00143829
O76036	NCR1	0.741569652	0.755075196	5.621E-06	0.001836735	2.8209E-05	0.00212828
P02649	APOE	1.325399966	1.282131162	2.048E-07	0.001137026	0.000283	0.001622935
P10721	KIT	1.489125587	1.416727689	7.463E-09	0.000719145	9.22E-06	0.001652089
P09758	TACSTD2	1.420541839	1.416748142	7.103E-08	0.000952381	0.00034927	0.001506317
P00533	EGFR	1.30263973	1.283543266	6.012E-06	0.001895044	1.9617E-05	0.001399417
Q9BYF1	ACE2	1.343109036	1.351129252	7.681E-07	0.00133139	0.00036686	0.006180758
P02649	APOE	1.244447984	1.206073828	7.474E-06	0.001924198	0.0024964	0.001622935
P02649	APOE	1.286022867	1.239419842	2.083E-06	0.001574344	0.00025421	0.001622935
P17931	LGALS3	0.752839331	0.772008464	2.628E-07	0.001156463	1.0971E-05	0.000398445
P35475	IDUA	1.411818675	1.445766652	5.303E-07	0.001253644	7.8693E-05	0.000748299
P01374, Q06643	LTA LTB	1.393305269	1.389026313	1.467E-06	0.001477162	9.2873E-05	0.004664723
P29965	CD40LG	1.474153245	1.453863625	1.945E-06	0.00154519	0.00291881	0.003790087

Uniprot ID	Gene ID	Fold expression	Predicted Linear Model Ratios (IPF/Healthy)	P-values significant after Bonferroni correction	FDR	Age-adjusted P-values significant after Bonferroni Correction	Age-adjusted FDR
Q9Y4X3	CCL27	1.173722536	1.162169851	3.663E-06	0.001681244	6.6273E-05	0.00122449
P07585	DCN	1.20880279	1.189205245	5.211E-06	0.001817298	0.00010196	0.001885326
Q9NP95	FGF20	1.438391531	1.407985353	5.916E-06	0.001875607	0.00576751	0.002390671
P08684	CYP3A4	1.445797436	1.410315476	3.527E-06	0.001661808	5.9022E-05	0.00111759
P29279	CTGF	1.32159639	1.299436776	5.526E-06	0.001827017	0.00012742	0.002585034
P56470	LGALS4	1.363080837	1.386548727	2.305E-07	0.001146744	0.0004329	0.001642371
Q15582	TGFBI	1.377537653	1.384270242	1.785E-06	0.001525753	0.00011283	0.001273081
Q8IWV2	CNTN4	1.266326604	1.257256862	4.96E-06	0.001797862	1.4765E-05	0.001107872
O94779	CNTN5	1.258655438	1.254045937	1.594E-06	0.001496599	8.4023E-05	0.002837707
P06493, P14635	CDC2 CCNB1	1.198173718	1.181337678	3.301E-06	0.001613217	0.00089827	0.005578231
Q92876	KLK6	0.864513016	0.866147653	2.492E-06	0.001603499	0.00035015	0.006375121
O00626	CCL22	1.424081215	1.429480123	3.309E-06	0.001622935	0.00020088	0.004635569
P01282	VIP	1.424445332	1.413234053	5.966E-06	0.001885326	0.00344855	0.002089407
Q6UXD5	SEZ6L2	1.407069992	1.369992374	8.816E-06	0.001972789	0.0005441	0.009193392
P68036	UBE2L3	1.467831972	1.427595832	3.606E-06	0.001671526	0.00063943	0.009115646
Q96GD0	PDXP	1.490673622	1.447598284	7.077E-06	0.00191448	0.00047606	0.000835763
P08620	FGF4	1.23892107	1.229893786	6.193E-07	0.001302235	0.00118849	0.001661808
Q99075	HBEGF	1.293217012	1.291360122	4.647E-07	0.00122449	0.00106179	0.000758017
P20783	NTF3	1.414871631	1.405958216	5.997E-07	0.001292517	0.00121293	0.000456754
P32004	L1CAM	1.422955729	1.402037028	4.477E-07	0.001214772	1.0997E-05	0.002361516
O43323	DHH	1.427120242	1.403360929	1.827E-06	0.001535471	0.00075255	0.000864917
O43320	FGF16	1.401626175	1.370275971	1.549E-06	0.00148688	0.00060477	0.002419825
O75356	ENTPD5	1.27036547	1.282597991	3.47E-06	0.001652089	1.6184E-05	0.001020408
Q4KMG0	CDON	1.438856529	1.395132851	6.493E-06	0.001904762	1.3063E-05	0.000573372
P10909	CLU	1.268085243	1.273973382	4.497E-06	0.001768707	9.0924E-05	0.000826045
Q9NZU1	FLRT1	1.438468618	1.404044634	9.319E-07	0.001360544	0.00032809	0.002069971
P21217	FUT3	1.457634251	1.441771268	2.029E-07	0.001127308	0.00057164	0.000874636
Q12884	FAP	1.355855033	1.36028669	3.434E-06	0.001642371	0.00025698	0.000136054
Q02241	KIF23	1.439636723	1.387282427	3.31E-06	0.001632653	0.00061253	0.000417881
P45984	MAPK9	1.366287937	1.385188001	5.896E-06	0.001865889	0.00016857	0.001302235

Uniprot ID	Gene ID	Fold expression	Predicted Linear Model Ratios (IPF/Healthy)	P-values significant after Bonferroni correction	FDR	Age-adjusted P-values significant after Bonferroni Correction	Age-adjusted FDR
P48061	CXCL12	0.779402388	0.782938513	9.425E-06	0.001982507	6.6164E-06	0.00447036
P29401	TKT	0.76346032	0.750233644	0.0004592	0.003002915	7.2265E-06	0.009737609

Upregulated in IPF AND Age-adjusted AND non-age-adjusted significant
Upregulated in IPF AND non-age significant
Age-adjusted AND/OR non-age-adjusted significant but not biologically relevant
Downregulated in IPF AND Age-adjusted AND non-age-adjusted significant
Downregulated in IPF AND Age-adjusted significant
Downregulated in IPF AND non-age-adjusted significant

Table S6 ClueGO analysis of biological roles of upregulated proteins in IPF plasma

GOTerm	Ontology Source	Term PValue	Term PValue Corrected with Bonferroni	Group PValue Corrected with Bonferroni	% Associated Genes	Nr. Genes	Associated Genes Found
regulation of cardiac muscle hypertrophy	GO_BiologicalProcess-GOA_09.02.2016_16h18	71.0E-9	2.9E-6	210.0E-9	12.82	5.00	[ECE1, GSK3A, GSK3B, PDE5A, PRKCA]
ErbB signaling pathway	KEGG_10.02.2016	4.0E-9	160.0E-9	17.0E-15	8.05	7.00	[GSK3B, MAP2K4, PIK3CA, PIK3R1, PRKCA, PRKCB, SRC]
Sphingolipid signaling pathway	KEGG_10.02.2016	950.0E-9	39.0E-6	17.0E-15	5.00	6.00	[PIK3CA, PIK3R1, PRKCA, PRKCB, RAC1, SPHK1]
VEGF signaling pathway	KEGG_10.02.2016	5.1E-12	210.0E-12	17.0E-15	13.11	8.00	[MAPKAPK2, PIK3CA, PIK3R1, PRKCA, PRKCB, RAC1, SPHK1, SRC]
B cell receptor signaling pathway	KEGG_10.02.2016	49.0E-9	2.0E-6	17.0E-15	8.22	6.00	[BTK, GSK3B, PIK3CA, PIK3R1, PRKCB, RAC1]
Fc epsilon RI signaling pathway	KEGG_10.02.2016	32.0E-9	1.3E-6	17.0E-15	8.82	6.00	[BTK, MAP2K4, PIK3CA, PIK3R1, PRKCA, RAC1]
Fc gamma R-mediated phagocytosis	KEGG_10.02.2016	210.0E-9	8.6E-6	17.0E-15	6.45	6.00	[PIK3CA, PIK3R1, PRKCA, PRKCB, RAC1, SPHK1]
Thyroid hormone signaling pathway	KEGG_10.02.2016	860.0E-9	35.0E-6	17.0E-15	5.08	6.00	[GSK3B, PIK3CA, PIK3R1, PRKCA, PRKCB, SRC]
AGE-RAGE signaling pathway in diabetic complications	KEGG_10.02.2016	340.0E-9	14.0E-6	17.0E-15	5.94	6.00	[FN1, PIK3CA, PIK3R1, PRKCA, PRKCB, RAC1]
Bacterial invasion of epithelial cells	KEGG_10.02.2016	2.4E-6	99.0E-6	17.0E-15	6.41	5.00	[FN1, PIK3CA, PIK3R1, RAC1, SRC]
T cell costimulation	GO_ImmuneSystemProcess-GOA_09.02.2016_16h18	210.0E-9	8.6E-6	17.0E-15	6.45	6.00	[CSK, PIK3CA, PIK3R1, RAC1, SRC, TNFSF13B]
platelet activation	GO_BiologicalProcess-GOA_09.02.2016_16h18	430.0E-15	17.0E-12	17.0E-15	4.69	13.00	[APOA1, CLIC1, CSK, F5, FN1, PIK3CA, PIK3R1, PRKCA, PRKCB, RAC1, SELP, SRC, TEC]
regulation of cellular response to insulin stimulus	GO_BiologicalProcess-GOA_09.02.2016_16h18	700.0E-9	28.0E-6	17.0E-15	8.20	5.00	[GSK3A, PIK3R1, PRKCA, PRKCB, SRC]
Complement and coagulation cascades	KEGG_10.02.2016	1.3E-6	53.0E-6	3.9E-6	7.25	5.00	[C1R, C4B, C5, F5, SERPINA5]

Table S7 **ClueGo analysis of the biological roles of downregulated proteins in IPF plasma**

GOTerm	Ontology Source	Term PValue	Term PValue Corrected with Bonferroni	Group PValue Corrected with Bonferroni	% Associated Genes	Nr. Genes	Associated Genes Found
acute inflammatory response	GO_BiologicalProcess-GOA_09.02.2016_16h18	67.0E-9	11.0E-6	740.0E-9	6.10	10.00	[C3, CRP, F3, GSTP1, LBP, PTGS2, SAA1, SERPINA3, SERPING1, TIMP1]
Fc receptor signaling pathway	GO_ImmuneSystemProcess-GOA_09.02.2016_16h18	650.0E-12	110.0E-9	3.4E-15	4.09	17.00	[CAMK2B, CAMK2D, CDKN1B, CFL1, CSF2, FYN, IL2, IL3, MAP2K1, MAP2K2, PEBP1, PSMA2, PTPN11, RPS27A, SHC1, UBE2N, YES1]
response to peptide hormone	GO_BiologicalProcess-GOA_09.02.2016_16h18	10.0E-15	1.8E-12	3.4E-15	4.07	26.00	[ANXA1, CAMK2B, CAMK2D, CDKN1B, CSF2, FYN, GOT1, GSTP1, IGFBP1, IGFBP2, IL2, IL3, MAP2K1, MAP2K2, NAMPT, PEBP1, POR, PSMA2, PTPN1, PTPN11, PTPN6, RETN, RPS27A, SHC1, TFF3, TIMP1]
cellular response to fibroblast growth factor stimulus	GO_BiologicalProcess-GOA_09.02.2016_16h18	4.1E-9	720.0E-9	3.4E-15	4.26	15.00	[CAMK2B, CAMK2D, CDKN1B, CSF2, CXCL8, FYN, IL2, IL3, MAP2K1, MAP2K2, PEBP1, PSMA2, PTPN11, RPS27A, SHC1]
response to insulin	GO_BiologicalProcess-GOA_09.02.2016_16h18	11.0E-12	1.9E-9	3.4E-15	4.18	20.00	[CAMK2B, CAMK2D, CSF2, FYN, GOT1, GSTP1, IGFBP1, IGFBP2, IL2, IL3, MAP2K1, MAP2K2, NAMPT, PEBP1, PSMA2, PTPN1, PTPN11, RETN, RPS27A, SHC1]
Fc-epsilon receptor signaling pathway	GO_ImmuneSystemProcess-GOA_09.02.2016_16h18	3.8E-9	660.0E-9	3.4E-15	4.29	15.00	[CAMK2B, CAMK2D, CDKN1B, CSF2, FYN, IL2, IL3, MAP2K1, MAP2K2, PEBP1, PSMA2, PTPN11, RPS27A, SHC1, UBE2N]
cellular response to insulin stimulus	GO_BiologicalProcess-GOA_09.02.2016_16h18	97.0E-12	17.0E-9	3.4E-15	4.27	18.00	[CAMK2B, CAMK2D, CSF2, FYN, GOT1, GSTP1, IGFBP1, IL2, IL3, MAP2K1, MAP2K2, NAMPT, PEBP1, PSMA2, PTPN1, PTPN11, RPS27A, SHC1]
insulin receptor signaling pathway	GO_BiologicalProcess-GOA_09.02.2016_16h18	380.0E-12	66.0E-9	3.4E-15	4.61	16.00	[CAMK2B, CAMK2D, CSF2, FYN, IGFBP1, IL2, IL3, MAP2K1, MAP2K2, NAMPT, PEBP1, PSMA2, PTPN1, PTPN11, RPS27A, SHC1]
epidermal growth factor receptor signaling pathway	GO_BiologicalProcess-GOA_09.02.2016_16h18	150.0E-12	27.0E-9	3.4E-15	4.49	17.00	[CAMK2B, CAMK2D, CDKN1B, CSF2, FYN, IL2, IL3, ITGA1, MAP2K1, MAP2K2, MMP9, PEBP1, PLAUR, PSMA2, PTPN11, RPS27A, SHC1]
phagocytosis	GO_BiologicalProcess-GOA_09.02.2016_16h18	160.0E-9	28.0E-6	1.8E-6	4.26	12.00	[AIF1, ANXA1, C3, CFL1, CRP, FCN1, FYN, HMGB1, LBP, PRTN3, SFTPD, YES1]
peptidase regulator activity	GO_MolecularFunction-GOA_09.02.2016_16h18	150.0E-9	26.0E-6	14.0E-12	4.85	11.00	[C3, C4B_2, CAST, CDKN1B, CST3, PEBP1, PI3, SERPINA3, SERPING1, TIMP1, TNFSF14]
endopeptidase inhibitor activity	GO_MolecularFunction-GOA_09.02.2016_16h18	160.0E-9	28.0E-6	14.0E-12	5.56	10.00	[C3, C4B_2, CAST, CST3, PEBP1, PI3, SERPINA3, SERPING1, TIMP1, TNFSF14]
regulation of endopeptidase activity	GO_BiologicalProcess-GOA_09.02.2016_16h18	490.0E-15	86.0E-12	14.0E-12	4.96	20.00	[C3, C4B_2, CAST, CDKN1B, CST3, F3, GPI, HMGB1, HTRA2, MMP9, PARK7, PEBP1, PI3, PLAUR, POR, S100A9, SERPINA3, SERPING1,

TIMP1, TNFSF14]

GOTerm	Ontology Source	Term PValue	Term PValue Corrected with Bonferroni	Group PValue Corrected with Bonferroni	% Associated Genes	Nr. Genes	Associated Genes Found
negative regulation of endopeptidase activity	GO_BiologicalProcess-GOA_09.02.2016_16h18	69.0E-12	12.0E-9	14.0E-12	5.73	15.00	[C3, C4B_2, CAST, CST3, GPI, MMP9, PARK7, PEBP1, PI3, PLAUR, POR, SERPINA3, SERPING1, TIMP1, TNFSF14]
regulation of cysteine-type endopeptidase activity involved in apoptotic process	GO_BiologicalProcess-GOA_09.02.2016_16h18	110.0E-9	20.0E-6	14.0E-12	4.98	11.00	[CDKN1B, F3, GPI, HMGB1, HTRA2, MMP9, PARK7, PLAUR, POR, S100A9, TNFSF14]
positive regulation of leukocyte activation	GO_ImmuneSystemProcess-GOA_09.02.2016_16h18	21.0E-9	3.8E-6	25.0E-9	4.12	14.00	[AIF1, ANXA1, FYN, HAVCR2, HMGB1, IGFBP2, IL2, LBP, PTPN11, PTPN6, S100A9, TIMP1, TNFSF14, YES1]
leukocyte proliferation	GO_BiologicalProcess-GOA_09.02.2016_16h18	4.0E-9	710.0E-9	25.0E-9	4.70	14.00	[AIF1, ANXA1, CSF1, FYN, GSTP1, HMGB1, IGFBP2, IL2, IL3, PTPN6, S100A9, SFTPD, TIMP1, TNFSF14]
mononuclear cell proliferation	GO_BiologicalProcess-GOA_09.02.2016_16h18	180.0E-9	32.0E-6	25.0E-9	4.21	12.00	[AIF1, ANXA1, CSF1, FYN, HMGB1, IGFBP2, IL2, PTPN6, S100A9, SFTPD, TIMP1, TNFSF14]
T cell proliferation	GO_ImmuneSystemProcess-GOA_09.02.2016_16h18	280.0E-9	49.0E-6	25.0E-9	5.24	10.00	[AIF1, ANXA1, FYN, HMGB1, IGFBP2, IL2, PTPN6, SFTPD, TIMP1, TNFSF14]
regulation of T cell activation	GO_ImmuneSystemProcess-GOA_09.02.2016_16h18	83.0E-9	14.0E-6	25.0E-9	4.06	13.00	[AIF1, ANXA1, FYN, HAVCR2, HMGB1, IGFBP2, IL2, PTPN11, PTPN6, SFTPD, TIMP1, TNFSF14, YES1]
positive regulation of T cell activation	GO_ImmuneSystemProcess-GOA_09.02.2016_16h18	150.0E-9	26.0E-6	25.0E-9	4.85	11.00	[AIF1, ANXA1, FYN, HMGB1, IGFBP2, IL2, PTPN11, PTPN6, TIMP1, TNFSF14, YES1]
regulation of leukocyte proliferation	GO_BiologicalProcess-GOA_09.02.2016_16h18	11.0E-9	1.9E-6	25.0E-9	5.43	12.00	[AIF1, ANXA1, CSF1, GSTP1, HMGB1, IGFBP2, IL2, IL3, PTPN6, S100A9, SFTPD, TIMP1]
positive regulation of leukocyte proliferation	GO_BiologicalProcess-GOA_09.02.2016_16h18	250.0E-9	44.0E-6	25.0E-9	6.25	9.00	[AIF1, ANXA1, CSF1, HMGB1, IGFBP2, IL2, IL3, S100A9, TIMP1]
cellular response to interferon-gamma	GO_ImmuneSystemProcess-GOA_09.02.2016_16h18	280.0E-9	50.0E-6	150.0E-12	6.16	9.00	[AIF1, CAMK2B, CAMK2D, CCL14, CCL23, PTPN1, PTPN11, PTPN6, SYNCRIP]
ERK1 and ERK2 cascade	GO_BiologicalProcess-GOA_09.02.2016_16h18	91.0E-12	15.0E-9	150.0E-12	5.62	15.00	[C3, CAMK2D, CCL14, CCL23, EPHA2, GSTP1, HMGB1, MAP2K1, MAP2K2, PLA2G2A, PTPN1, PTPN11, PTPN6, S100A9, TIMP1]
regulation of ERK1 and ERK2 cascade	GO_BiologicalProcess-GOA_09.02.2016_16h18	4.3E-9	750.0E-9	150.0E-12	5.22	13.00	[C3, CAMK2D, CCL14, CCL23, EPHA2, GSTP1, HMGB1, PLA2G2A, PTPN1, PTPN11, PTPN6, S100A9, TIMP1]
granulocyte chemotaxis	GO_ImmuneSystemProcess-GOA_09.02.2016_16h18	2.0E-9	350.0E-9	22.0E-9	8.77	10.00	[ANXA1, CCL14, CCL23, CSF1, CXCL8, DAPK2, ITGA1, LBP, S100A9, SAA1]
neutrophil chemotaxis	GO_ImmuneSystemProcess-GOA_09.02.2016_16h18	120.0E-9	21.0E-6	22.0E-9	8.42	8.00	[CCL14, CCL23, CXCL8, DAPK2, ITGA1, LBP, S100A9, SAA1]
positive regulation of leukocyte chemotaxis	GO_ImmuneSystemProcess-GOA_09.02.2016_16h18	55.0E-9	9.7E-6	74.0E-24	9.30	8.00	[AIF1, CSF1, CXCL11, CXCL8, DAPK2, HMGB1, LBP, TNFSF14]

GOTerm	Ontology Source	Term PValue	Term PValue Corrected with Bonferroni	Group PValue Corrected with Bonferroni	% Associated Genes	Nr. Genes	Associated Genes Found
inflammatory response	GO_BiologicalProcess-GOA_09.02.2016_16h18	26.0E-18	4.6E-15	74.0E-24	4.20	30.00	[AIF1, ANXA1, C3, C4B_2, CCL14, CCL23, CMA1, CRP, CSF1, CTSS, CXCL11, CXCL8, F3, GSTP1, HAVCR2, HMGB1, IL2, LBP, MAPK13, OLR1, PARK7, PGLYRP1, PLA2G2A, PTGS2, S100A9, SAA1, SERPINA3, SERPING1, TIMP1, TNFRSF1B]
positive regulation of response to external stimulus	GO_BiologicalProcess-GOA_09.02.2016_16h18	20.0E-18	3.6E-15	74.0E-24	7.03	22.00	[AIF1, C3, CCL14, CCL23, CSF1, CTSS, CXCL11, CXCL8, DAPK2, F3, HAVCR2, HMGB1, IL16, IL2, LBP, MAPK13, PARK7, PLA2G2A, PTGS2, S100A9, SCARF1, TNFSF14]
positive regulation of defense response	GO_BiologicalProcess-GOA_09.02.2016_16h18	4.9E-12	860.0E-12	74.0E-24	4.38	20.00	[C3, CCL14, CCL23, CTSS, FCN1, FYN, HAVCR2, HMGB1, IL2, LBP, LTF, MAP2K1, MAPK13, PGLYRP1, PLA2G2A, PSMA2, PTGS2, RPS27A, S100A9, UBE2N]
positive regulation of chemotaxis	GO_BiologicalProcess-GOA_09.02.2016_16h18	9.7E-9	1.7E-6	74.0E-24	7.46	10.00	[AIF1, CSF1, CXCL11, CXCL8, DAPK2, F3, HMGB1, IL16, LBP, TNFSF14]
regulation of response to wounding	GO_BiologicalProcess-GOA_09.02.2016_16h18	23.0E-18	4.1E-15	74.0E-24	5.59	25.00	[ANXA1, ANXA2, C3, CCL14, CCL23, CMA1, CTSS, F3, GSTP1, HMGB1, IL2, LBP, MAP2K1, MAP2K2, MAPK13, PARK7, PGLYRP1, PLA2G2A, PLAUR, PTGS2, S100A9, SAA1, SCARF1, SERPING1, TNFRSF1B]
positive regulation of response to wounding	GO_BiologicalProcess-GOA_09.02.2016_16h18	3.0E-12	520.0E-12	74.0E-24	8.09	14.00	[ANXA1, C3, CCL14, CCL23, CTSS, F3, HMGB1, IL2, LBP, MAPK13, PLA2G2A, PTGS2, S100A9, SCARF1]
regulation of inflammatory response	GO_BiologicalProcess-GOA_09.02.2016_16h18	800.0E-15	140.0E-12	74.0E-24	5.70	18.00	[ANXA1, C3, CCL14, CCL23, CMA1, CTSS, GSTP1, IL2, LBP, MAPK13, PARK7, PGLYRP1, PLA2G2A, PTGS2, S100A9, SAA1, SERPING1, TNFRSF1B]
positive regulation of inflammatory response	GO_BiologicalProcess-GOA_09.02.2016_16h18	3.9E-9	680.0E-9	74.0E-24	8.20	10.00	[C3, CCL14, CCL23, CTSS, IL2, LBP, MAPK13, PLA2G2A, PTGS2, S100A9]
TNF signaling pathway	KEGG_10.02.2016	380.0E-9	66.0E-6	4.2E-6	7.27	8.00	[CASP10, CSF1, CSF2, MAP2K1, MAPK13, MMP9, PTGS2, TNFRSF1B]
Proteoglycans in cancer	KEGG_10.02.2016	48.0E-9	8.5E-6	530.0E-9	5.42	11.00	[CAMK2B, CAMK2D, ITGB1, MAP2K1, MAP2K2, MAPK13, MMP9, MSN, PLAUR, PTPN11, PTPN6]
positive regulation of tyrosine phosphorylation of Stat5 protein	GO_BiologicalProcess-GOA_09.02.2016_16h18	110.0E-9	20.0E-6	140.0E-15	25.00	5.00	[CSF2, FYN, IL2, IL3, TIMP1]
cytokine activity	GO_MolecularFunction-GOA_09.02.2016_16h18	1.1E-12	190.0E-12	140.0E-15	6.81	16.00	[CCL14, CCL23, CSF1, CSF2, CXCL11, CXCL8, GDF5, GPI, HMGB1, IL16, IL2, IL3, NAMPT, S100A9, TIMP1, TNFSF14]
cytokine receptor binding	GO_MolecularFunction-GOA_09.02.2016_16h18	31.0E-9	5.4E-6	140.0E-15	4.42	13.00	[CCL14, CCL23, CSF1, CSF2, CXCL11, CXCL8, GDF5, IL2, IL3, S100A9, SHC1, TIMP1,

							TNFSF14]

GOTerm	Ontology Source	Term PValue	Term PValue Corrected with Bonferroni	Group PValue Corrected with Bonferroni	% Associated Genes	Nr. Genes	Associated Genes Found
peptidyl-tyrosine phosphorylation	GO_BiologicalProcess-GOA_09.02.2016_16h18	5.5E-9	970.0E-9	140.0E-15	4.17	15.00	[CSF2, DDR2, EPHA2, FYN, IL2, IL3, ITGB1, MAP2K1, MAP2K2, PTPN1, PTPN6, S100A9, SHC1, TIMP1, YES1]
regulation of peptidyl-tyrosine phosphorylation	GO_BiologicalProcess-GOA_09.02.2016_16h18	210.0E-9	37.0E-6	140.0E-15	4.68	11.00	[CSF2, FYN, IL2, IL3, ITGB1, PTPN1, PTPN6, S100A9, SHC1, TIMP1, YES1]

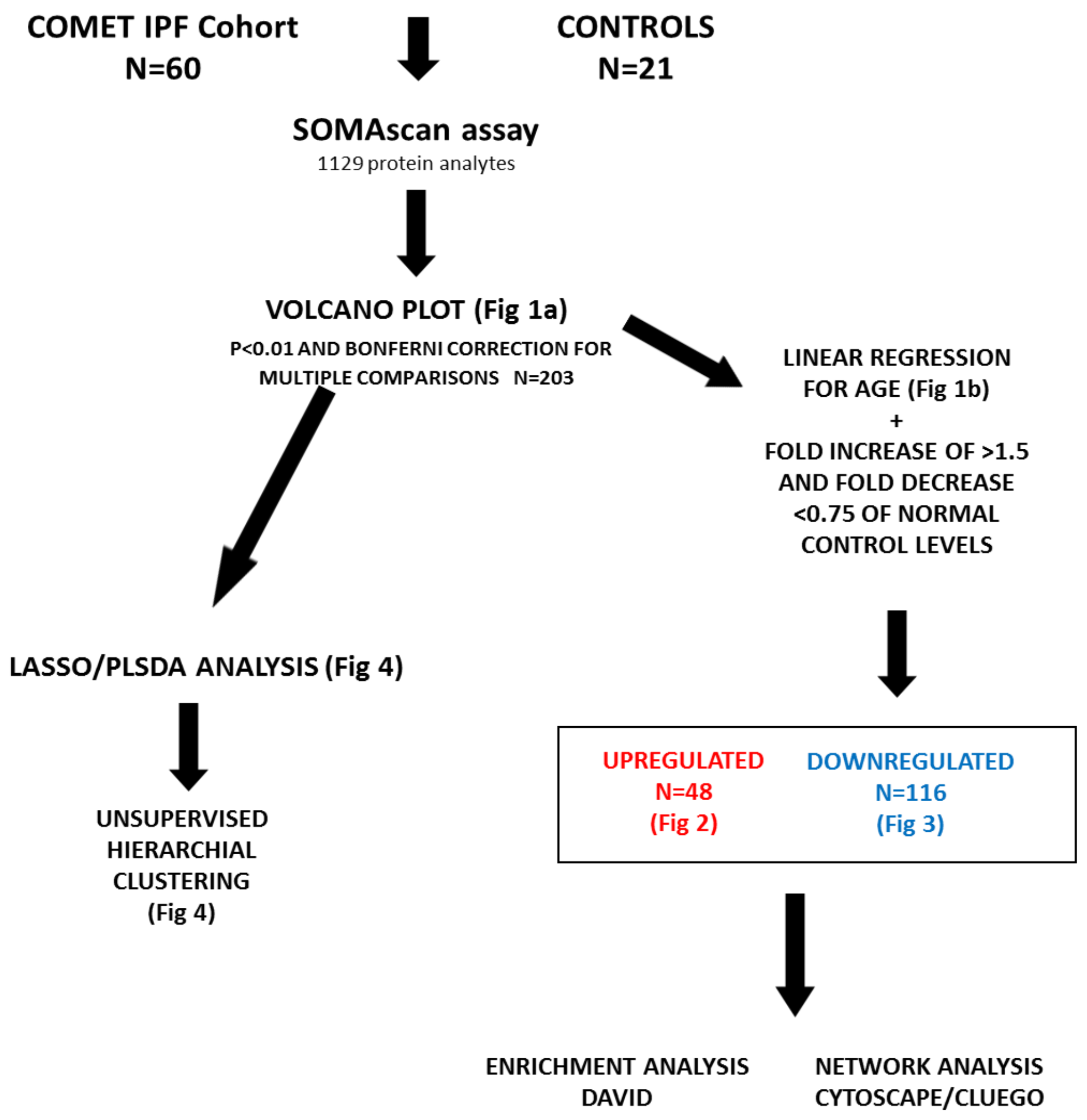

Figure S1 **Schematic representation of approach adopted in this study.**

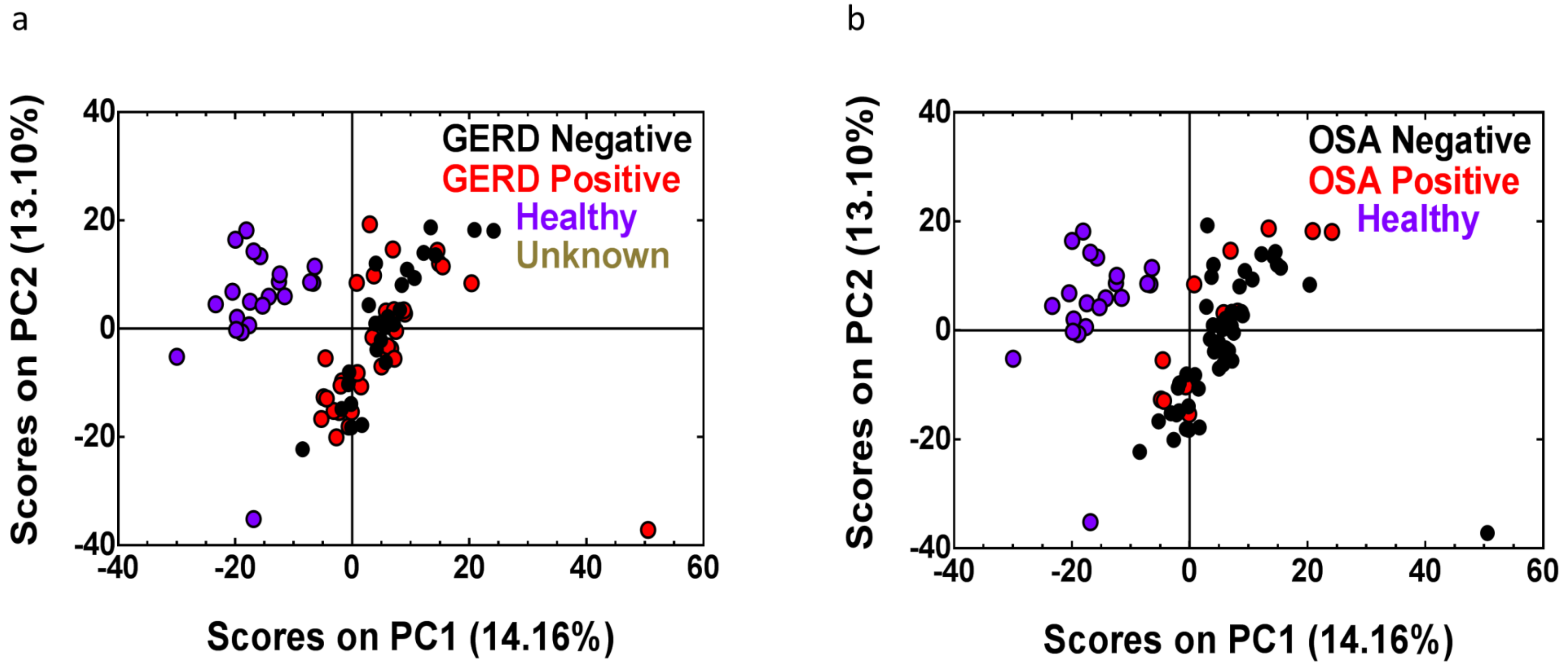

Figure S2. Principal Component Analysis (PCA) showed that the largest, unbiased difference in the IPF-Healthy dataset is between the IPF and healthy groups, with comorbidities in the IPF patients having little effect. (a) A PCA model based on all 1129 measured blood proteins captured 27.26% of the total variance in the data, with PC1 explaining 14.16% of the variance and PC2, 13.10%. In this model, the healthy patients score in the negative region of PC1, and the IPF patients score mostly in the positive area of PC1. IPF patients with GERD do not cluster together within the IPF group; these patients are mixed evenly with the IPF patients who do not have GERD. **(b)** Similarly, when looking at IPF patients with obstructive sleep apnea (OSA), it can be seen that these patients are spread throughout the IPF grouping in the PCA and do not form their own cluster. The main difference in this PCA model is still between the healthy and IPF patients.

	regulation of cardiac muscle hypertrophy	platelet activation	T cell costimulation	regulation of cellular response to insulin stimulus	ErbB signaling pathway	Sphingolipid signaling pathway	VEGF signaling pathway	Complement and coagulation cascades	B cell receptor signaling pathway	Fc epsilon RI signaling pathway	Fc gamma R-mediated phagocytosis	Thyroid hormone signaling pathway	AGE-RAGE signaling pathway in diabetic complications	Bacterial invasion of epithelial cells
regulation of cardiac muscle hypertrophy	1.00	-0.23	-0.27	0.26	0.14	-0.04	-0.11	-0.24	-0.04	-0.04	-0.04	0.19	-0.04	-0.24
platelet activation	-0.23	1.00	0.31	0.23	0.23	0.31	0.31	-0.23	0.15	0.15	0.31	0.31	0.46	0.38
T cell costimulation	-0.27	0.31	1.00	0.19	0.28	0.35	0.42	-0.27	0.35	0.35	0.35	0.35	0.35	0.65
regulation of cellular response to insulin stimulus	0.26	0.23	0.19	1.00	0.57	0.42	0.50	-0.24	0.19	0.19	0.42	0.65	0.42	0.26
ErbB signaling pathway	0.14	0.23	0.28	0.57	1.00	0.49	0.53	-0.29	0.49	0.49	0.49	0.90	0.49	0.36
Sphingolipid signaling pathway	-0.04	0.31	0.35	0.42	0.49	1.00	0.81	-0.27	0.57	0.57	1.00	0.57	0.78	0.42
VEGF signaling pathway	-0.11	0.31	0.42	0.50	0.53	0.81	1.00	-0.31	0.42	0.42	0.81	0.61	0.61	0.50
Complement and coagulation cascades	-0.24	-0.23	-0.27	-0.24	-0.29	-0.27	-0.31	1.00	-0.27	-0.27	-0.27	-0.27	-0.27	-0.24
B cell receptor signaling pathway	-0.04	0.15	0.35	0.19	0.49	0.57	0.42	-0.27	1.00	0.57	0.57	0.57	0.57	0.42
Fc epsilon RI signaling pathway	-0.04	0.15	0.35	0.19	0.49	0.57	0.42	-0.27	0.57	1.00	0.57	0.35	0.57	0.42
Fc gamma R-mediated phagocytosis	-0.04	0.31	0.35	0.42	0.49	1.00	0.81	-0.27	0.57	0.57	1.00	0.57	0.78	0.42
Thyroid hormone signaling pathway	0.19	0.31	0.35	0.65	0.90	0.57	0.61	-0.27	0.57	0.35	0.57	1.00	0.57	0.42
AGE-RAGE signaling pathway in diabetic complications	-0.04	0.46	0.35	0.42	0.49	0.78	0.61	-0.27	0.57	0.57	0.78	0.57	1.00	0.65
Bacterial invasion of epithelial cells	-0.24	0.38	0.65	0.26	0.36	0.42	0.50	-0.24	0.42	0.42	0.42	0.42	0.65	1.00

Figure S3 **Kappa statistics from the upregulated proteome.** Level of agreement between gene terms is measured by Kappa statistics(default <4). Red scale depicts level of agreement from very high (1) to very low (-1).

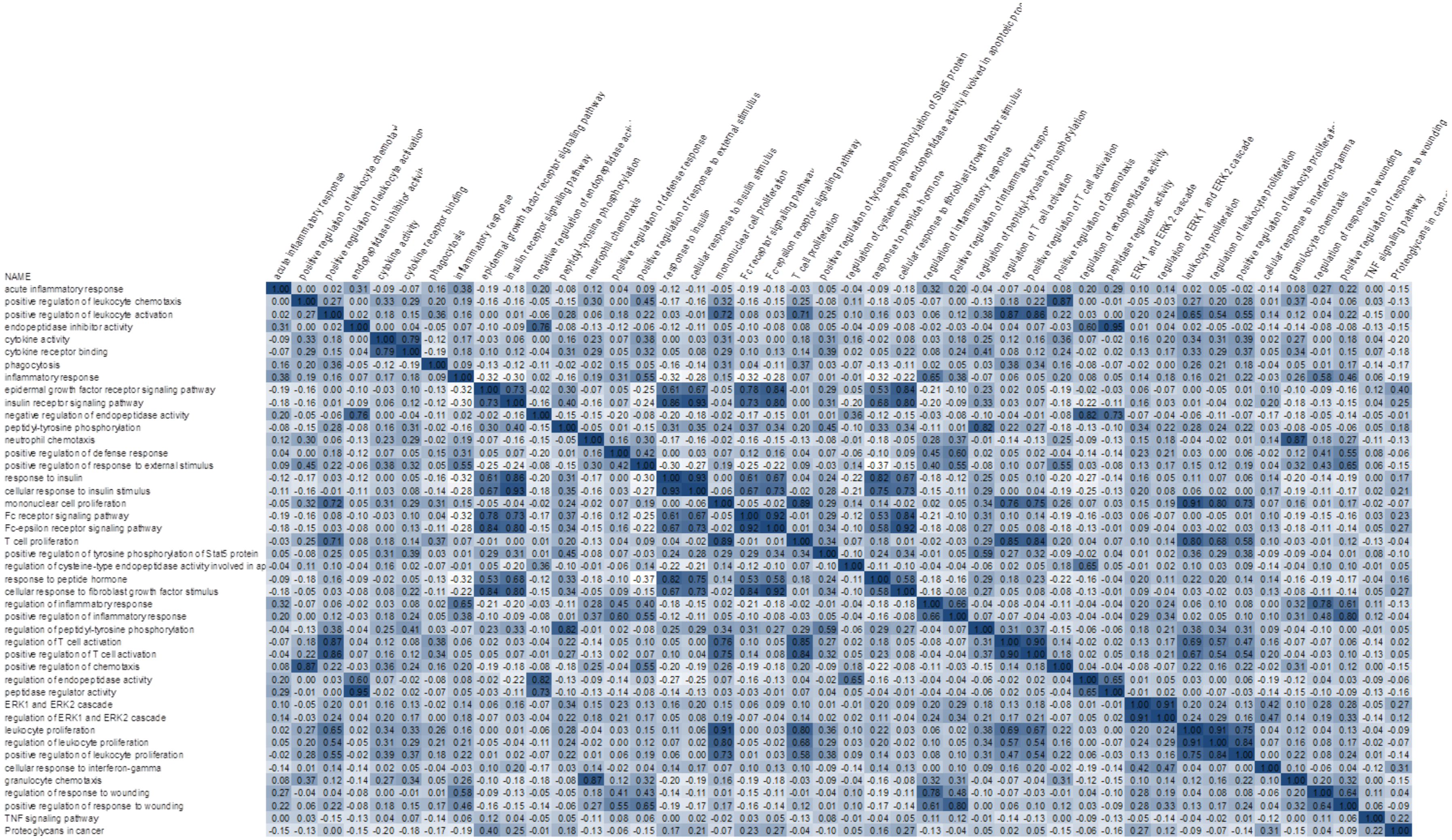

Figure S4 **Kappa statistics from the downregulated proteome.** Level of agreement between gene terms is measured by Kappa statistics(default <4). Red scale depicts level of agreement from very high (1) to very low (-1).

Figure S5 **DAVID analysis by GO Biological Process of the 8 protein identified LASSO signature**

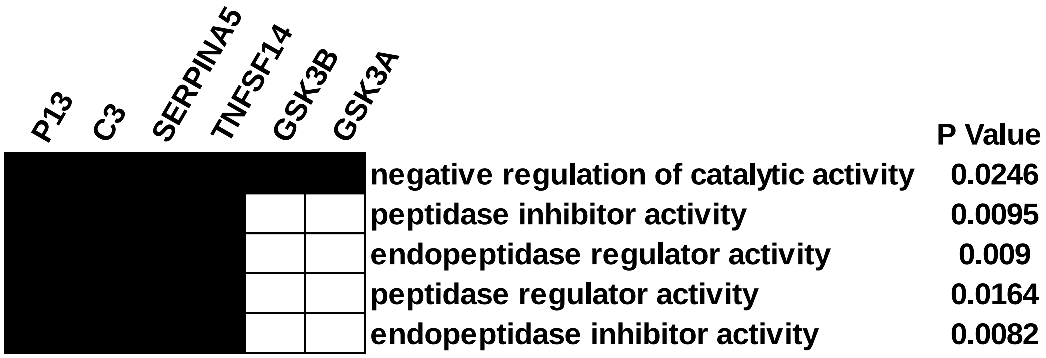

Supplement: Supplementary Information [file srep46560-s1.pdf]
